# A Comprehensive Evaluation of the Antibody-Verified Status of Eplets Listed in the HLA Epitope Registry

**DOI:** 10.3389/fimmu.2021.800946

**Published:** 2022-01-28

**Authors:** Suzanne Bezstarosti, Kim H. Bakker, Cynthia S. M. Kramer, Johan W. de Fijter, Marlies E. J. Reinders, Arend Mulder, Frans H. J. Claas, Sebastiaan Heidt

**Affiliations:** ^1^Department of Immunology, Leiden University Medical Center, Leiden, Netherlands; ^2^Department of Internal Medicine (Nephrology), Leiden University Medical Center, Leiden, Netherlands; ^3^Department of Internal Medicine, Erasmus Medical Center Transplantation Institute, University Medical Center Rotterdam, Rotterdam, Netherlands; ^4^Eurotransplant Reference Laboratory, Leiden, Netherlands

**Keywords:** eplet, epitope-matching, transplantation, human leukocyte antigen, antibody verification, reactivity pattern, monoclonal antibody

## Abstract

Matching strategies based on HLA eplets instead of HLA antigens in solid organ transplantation may not only increase the donor pool for highly sensitized patients, but also decrease the incidence of *de novo* donor-specific antibody formation. However, since not all eplets are equally capable of inducing an immune response, antibody verification is needed to confirm their ability to be bound by antibodies, such that only clinically relevant eplets are considered. The HLA Epitope Registry has documented all theoretically defined HLA eplets along with their antibody verification status and has been the foundation for many clinical studies investigating eplet mismatch in transplantation. The verification methods for eplets in the Registry range from polyclonal sera from multi- and uni-parous women to murine and human monoclonal antibodies (mAbs), and antibodies purified by adsorption and elution from sera of HLA immunized individuals. The classification of antibody verification based on different methods for validation is problematic, since not all approaches represent the same level of evidence. In this study, we introduce a classification system to evaluate the level of evidence for the antibody-verified status of all eplets in the HLA Epitope Registry. We demonstrate that for a considerable number of eplets, the antibody-verified status is solely based on polyclonal serum reactivity of multiparous women or on reactivity of murine mAbs. Furthermore, we noted that a substantial proportion of patient sera analyses and human mAb data presented in the HLA Epitope Registry Database has never been published in a peer-reviewed journal. Therefore, we tested several unpublished human HLA-specific mAbs by luminex single antigen beads assay to analyze their HLA reactivity for eplet antibody verification. Although the majority of analyzed mAbs indeed verified their assigned eplets, this was not the case for a number of eplets. This comprehensive overview of evidence for antibody verification of eplets in the HLA Epitope Registry is instrumental for future investigations towards eplet immunogenicity and clinical studies considering antibody-verified eplet mismatch in transplantation and warrants further standardization of antibody verification using high quality data.

## Introduction

Donor-specific antibodies (DSA) are formed against mismatched polymorphic amino acid residues on donor human leukocyte antigens (HLA) and are a major complication in renal transplantation, leading to chronic rejection and graft loss (1, 2). HLA eplets are small configurations of surface-exposed amino acids within a 3-3.5 Ångstrom (Å) radius (3, 4) and resemble the functional epitope, which generally determines the specificity of the antibody through interaction with the complementarity-determining region 3 (CDR3) of the heavy chain of the antibody (5–7). Consideration of HLA eplets instead of HLA antigens may not only refine HLA matching strategies, resulting in decreased DSA formation, but also expand the donor pool for highly sensitized patients and facilitate personalized immunosuppressive treatment based on immunological risk evaluation (8). Indeed, several studies have shown that eplet mismatches are correlated with DSA formation, graft rejection and graft loss (9–14). However, as eplets have been theoretically defined, their clinical relevance needs to be validated by antibody verification (8, 15). Although antibody-verified eplet mismatches have been demonstrated to correlate with DSA formation and graft survival (13, 14), recent reports also indicated that there are still clinically relevant eplets which have not been antibody-verified yet (13, 16).

The HLA Epitope Registry is an online database founded under auspices of the 16^th^ International HLA and Immunogenetics Workshop in 2012, which has documented all theoretically defined eplets, as well as their antibody verification status (17) with the aim to reflect the eplet repertoire incorporated in the widely used HLAMatchmaker software. The Registry has formed a pivotal source of information on eplets that have been defined on HLA as well as on MICA (Human Major Histocompatibility Complex Class I Chain-Related gene A) and has been of great benefit to the field of histocompatibility. Antibody-verified eplets in the Registry have been verified by analyzing reactivity patterns of either polyclonal sera from multi- or uni-parous women, murine monoclonal antibodies (mAb), human mAbs, or antibodies purified by adsorption and elution from sera of HLA immunized individuals (18, 19). The use of different methods for eplet verification is problematic because not all approaches represent the same level of evidence. In most, if not all cases, reactivity of polyclonal sera in luminex single antigen bead (SAB) assays cannot be attributed to a monoclonal response directed against a single eplet, and even adsorption and elution of antibodies from patient sera does not guarantee that the SAB reactivity is caused by antibody reactivity against a single eplet. Also the notion that purification of IgG may reveal ‘‘natural’’ (non-pathogenic) anti-HLA antibodies need to be considered when eluted antibodies are analyzed (20, 21). Additionally, the use of murine mAbs does not provide sufficient evidence for immunogenicity in the human setting, since the immunogenicity of mismatched HLA antigens is affected by the recipient’s HLA type (22). Consequently, murine mAbs may recognize different HLA epitopes than human antibodies. Therefore, we consider the use of human HLA-specific mAbs as the highest level of evidence for eplet antibody verification. In previous versions of the Registry, a subcategory of provisionally antibody-verified eplets was present, which unfortunately was discontinued. Such category is useful for data that hint towards true eplet-antibody interaction, but that are not strong enough for actual antibody verification.

The disparity in the level of evidence of antibody verification hampers the clinical application of evidence-based eplet matching and is not only caused by the different methods of antibody verification, but also by the incorporation of unpublished data in the HLA Epitope Registry, as opposed to experimental evidence from peer-reviewed literature. In this paper, we establish a comprehensive overview of the evidence for the antibody verification status of eplets included in the Registry by evaluating the level of evidence of different experimental methods using a classification system. Furthermore, we show previously unpublished SAB analyses of a number of human HLA-specific mAbs that are included in the Registry. We demonstrate that antibody-verified status of 45% of the eplets is based on analysis of polyclonal sera, murine mAbs or experiments with low resolution HLA typed cells and that several human mAbs have been wrongfully attributed to the verification of certain eplets. Our results illustrate the heterogenous and occasionally nontransparent methods of antibody verification and stress the importance of standardization of experimental procedures for antibody verification of HLA eplets.

## Materials and Methods

### Review of References in the HLA Epitope Registry Databases

HLA Epitope Registry HLA-ABC, HLA-DRB, HLA-DQ and HLA-DP databases were accessed on http://www.EpRegistry.com.br on 28 January 2021. All literature references for antibody-verified eplets present in these databases were reviewed for their level of evidence according to **Table 1**. Eplets with one or more references of A1 or A2 level were considered truly antibody-verified. Eplets with level B, C or D were classified as *provisionally* antibody-verified, a category of verification that was present in first report of the HLA Epitope Registry (18), but has been removed since the second update (23). Human mAb data presented in the database that had not been published in a peer-reviewed journal were considered as not sufficient for antibody verification. In order to provide a thorough overview of the antibody verification status of eplets, recent papers that provide evidence for antibody verification and which were not included in the HLA Epitope Registry at the moment of data extraction were also evaluated for their level of evidence.

**Table 1:** Level of evidence.

**A1**	Human monoclonal antibody + single antigen beads (SAB) assay, possibly supported by complement dependent cytotoxicity assay (CDC) with high resolution HLA typed cells (second field).
**A2**	Adsorption and elution studies + SAB assay, possibly supported by CDC with high resolution HLA typed cells.
**B**	Patient serum tested in SAB assay and/or CDC with high resolution HLA typed cells.
**C**	Human monoclonal antibody *or* adsorption and elution studies *or* patient sera tested with low resolution HLA typed cells only (first field or serological typing).
**D**	Any reactivity analysis with antibodies from other species (e.g. murine monoclonal antibody).

### HLA-Specific Human Monoclonal Antibodies

For a number of eplets, human mAb data presented in the Registry has not been published in a peer-reviewed journal. Therefore, these human mAbs which were previously produced by cloned B cell hetero-hybridomas derived from pregnancy immunized individuals (24–28), were tested in luminex SAB assay and subsequently analyzed for their HLA-specificity. IgG human mAbs were tested in the Lifecodes HLA class I or HLA class II SAB assay (Immucor, Stamford, CT, USA) according to the manufacturer’s instructions. For IgM mAbs, the PE-conjugated goat anti-human IgG was replaced with a PE-conjugated anti-human IgM detection antibody (One Lambda, Canoga Park, CA, USA) used in 1:100 dilution. All mAbs were tested at a concentration of 10 ug/ml (29), unless the neat sample concentration was below 10 ug/ml. **Supplementary Table 1** lists the alleles present in the SAB panel that was used. HLA antibody data were analyzed with Match It! Antibody software version 1.3.0 (Immucor). Results were expressed as background-corrected mean fluorescence intensity (MFI). Bead-specific cut-off based on raw MFI/lowest ranked antigen (LRA) (MFI/LRA) in combination with raw MFI >750 was utilized to assign positive beads. For some mAbs, the reactivity pattern was corroborated by testing with One Lambda SAB assay (LABscreen, One Lambda, Canoga Park, CA, USA).

### Lymphocytotoxicity

Lymphocytotoxicity data for mAbs VDK1D12, VN2F1, DMS4G2 and SN66E3 were obtained from the 13th International HLA and Immunogenetics Workshop. In this project, a panel of more than 800 second-field HLA-typed cells were tested in complement dependent cytotoxicity (CDC) assays in twelve laboratories worldwide (30). Only cells with a single SAB reactive allele were included for analysis. The average CDC score for each allele was calculated from the previously determined reactivity grades 1 (negative), 2 (doubtful positive), 4 (weakly positive), 6 (positive) and 8 (strongly positive). Lymphocytotoxicity data for mAbs DK1G8 and VIE6C10 were obtained from earlier performed CDC assays with second-field HLA typed cells, which were carried out as previously described with mAb FK5 (pan HLA class I) as positive control (31). The percentages of target cell lysis were converted to CDC scores (0-10% lysis: 1, 11-20%: 2, 21-50%: 4, 51-80%: 6 and >80%: 8) (32) and average scores for each allele were calculated.

### Reactivity Analysis of Human mAbs

HLA Epitope Mismatch Algorithm (HLA-EMMA) version 1.05 (33) was used to determine the solvent accessible amino acid mismatches between the HLA of the antibody producer and the mismatched HLA allele of the immunizer. In case of an ambiguous second field HLA typing, the most likely second field typing was selected based on a high resolution typed panel (n=1305) from Leiden, the Netherlands (http://www.allelefrequencies.net/pop6001c.asp?pop_id=0003257). If the immunizer was unknown, the specificity of the bead with the highest MFI in SAB assay was used to determine amino acid mismatches. Next, we determined whether these solvent accessible amino acid mismatches were uniquely shared by the reactive HLA alleles and absent on the non-reactive HLA alleles. In order to visualize amino acid positions and to establish whether amino acids were within 3-3.5 Å to form an eplet, the following HLA crystal structures were visualized in Swissviewer (34): Protein Data Bank (PBD) 1A6A, 1M6O, 1S9V, 1UVQ, 1X7Q, 1XR9, 3BO8, 3RL1, 3UTQ, 3WL9, 4U1H, 4Z7U, 5IND and 6PCL (downloaded from https://www.rcsb.org/on July 26, 2021). When HLA crystal structures were not available, modelled PBD structures were used; 3PL6, 3WEX, 4I5B, 4NT6 and 4Z7U (downloaded from https://www.phla3d.com.br/ on July 26, 2021). For HLAMatchmaker analysis, ABC Antibody Analysis Program V3.1 and DRDQDP Antibody Analysis Program v3.1 were used (http://www.epitopes.net/).

### Review of Eplet Definitions

For every eplet with the antibody-verified status in the HLA Epitope Registry that consisted of more than one polymorphic residue, it was determined whether the involved amino acids were indeed within 3-3.5 Å using Swissviewer (34). If not, antibody reactivity analysis for this eplet was repeated using SAB data from the referenced paper to identify uniquely shared residues. If multiple uniquely shared residues were identified that were not within 3-3.5 Å (eplet definition), the eplet was classified as ‘‘reactivity pattern’’ (see **Box 1**).

Box 1 **Definitions:**

**Functional epitope:** The functional epitope determines the specificity of the antibody through its interaction with the complementarity-determining region 3 (CDR3) of the heavy chain of the antibody.

**Eplet**: The definition of an eplet resembles the functional epitope and comprises the minimal amino acid configuration on the HLA-molecule that is needed to induce an antibody response. Involved residues must be within 3-3.5 Å.

**Structural epitope:** The structural epitope comprises all amino acids of the HLA-molecule that are involved in the binding to the antibody paratope and spans a radius of approximately 15 Å.

**Reactivity pattern**: In some cases, the SAB analysis of a human mAb yields multiple uniquely shared residues or multiple uniquely shared combinations of residues that are not within 3-3.5 Å, indicating that there are multiple possible eplets that could have induced the formation of the antibody. Often, these amino acids are simultaneously present on HLA alleles, which limits the possibilities of determining the actual eplet using SAB or cellular assays. However, the fact that the residues involved always occur together on these HLA alleles, also means that these residues can be regarded as a ‘’reactivity pattern’’ and can be used as a single entity in matching strategies and immunological risk assessment for the vast majority of transplant patients.

## Results

### Antibody Verification of HLA Class I Eplets by Human mAbs

**Figure 1:**
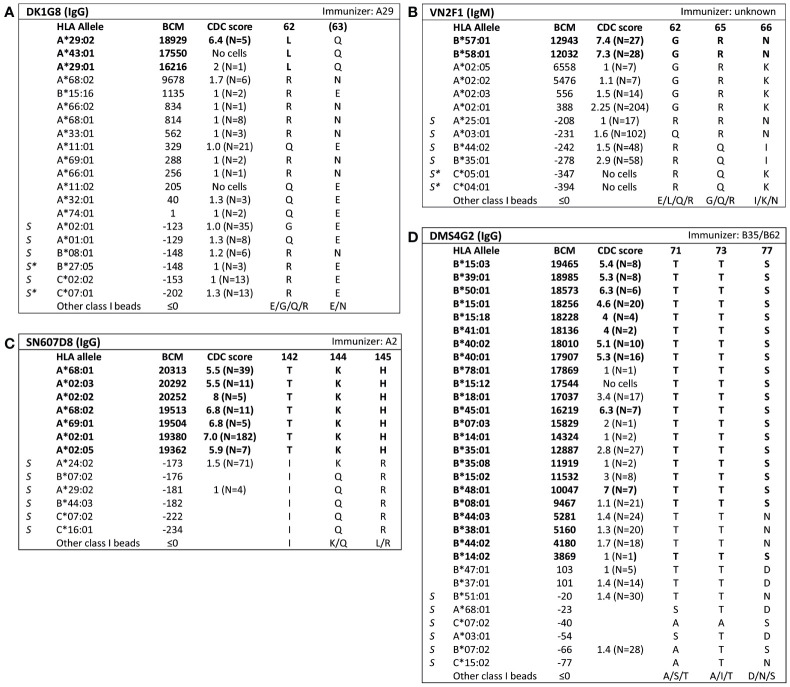
Comparison of the amino acid positions of interest of a selection of HLA class I alleles in the single antigen bead assay and complement dependent cytotoxicity assay for **(A)** mAb DK1G8, **(B)** VN2F1, **(C)** SN607D8 and **(D)** DMS4G2. mAb concentrations used for testing were 6.3 µg/ml for DMS4G2 and 10 µg/ml for the other mAbs. Self HLA alleles of the antibody producer marked with * are the most likely high resolution HLA typing due to ambiguous second-field typing. Alleles in bold are considered positive. Amino acid residues in bold are uniquely shared by the reactive alleles, or are part of a uniquely shared combination of residues. BCM, background corrected mean fluorescence intensity; CDC, complement dependent cytotoxicity; S, self HLA alleles of antibody producer.

For 13 HLA class I eplets in the HLA Epitope Registry, antibody-verified status was based on data of 15 mAbs that had not been published in a peer-reviewed journal. Therefore, these mAbs were re-tested in luminex SAB assay to determine whether they would indeed provide evidence for antibody verification of these eplets. SAB analysis of the mAbs was performed by comparison of amino acid sequences of the reactive alleles in SAB assay with non-reactive alleles to identify uniquely shared amino acids that could have induced the antibody response. These uniquely shared residues were then mapped to corresponding eplets (**Table 2**). Overall, 12 human mAbs indeed verified the eplet as listed in the HLA Epitope Registry. mAbs JOK3H4, OK2F3, VTM4D9, GK31F12, MUL6D1, GV2D5, VP5G3 and IND3H3 verified eplets 107W, 161D, 65QIA, 144QL, 151AHA, 163RG, 163RW and 65GK respectively (**Figures S1A–H**). For mAbs DK1G8 and VN2F1, SAB data did not only show several beads with MFI > 10,000 (uniquely shared by eplets 62LQ and 62GRN respectively), but also included multiple positive reactions with considerably lower MFIs (MFI 814 to 9678). Analysis of previously acquired CDC data demonstrated that cells bearing alleles with these lower MFI values were negative in CDC (**Figures 1A, B**). Thus, analysis of SAB and CDC data of mAbs DK1G8 and VN2F1 confirmed the verification of eplets 62LQ and 62GRN respectively. Analysis of mAb SN607D8, which is listed as evidence for antibody verification of eplet 144TKH (142T 144K 145H), showed that both 142T and 145H are uniquely shared residues and that 144K is not (**Figure 1C**). Therefore, it is possible that only one of these two residues, or the combination of 142T and 145H is required for antibody induction. However, since the combination of 142T, 144K and 145H is also uniquely shared and is within 3.5 Å (**Figure S2K**), it cannot be ruled out that all three residues are crucial. Therefore, we consider the SAB analysis of mAb SN607D8 as evidence for the antibody verification of eplet 144TKH. SAB data of mAb DMS4G2, which is listed in the HLA Epitope Registry for verification of eplet 71TTS, demonstrated a broad spectrum of positive MFI values and showed three alleles that do not bear the eplet but are positive in SAB (MFI 4180-5160) (**Figure 1D**). However, data from previously performed CDC assays demonstrated that these alleles were negative in CDC. Interestingly, also a number of alleles bearing eplet 71TTS were not reactive in CDC. Therefore, it appears that for this antibody producer, eplet 71TTS has induced the antibody response, but the antibody does not bind equally strong to all eplet-bearing alleles, presumably due to other amino acid residues that play a role in the antibody binding. For instance, although alleles B*14:01 and B*14:02 only have one amino acid mismatch on position 11 (non-exposed), there is a large difference in MFI (14324 vs 3869). We hypothesize that the different amino acid sequence influences the structural and electrostatic properties of the epitope and consequently alters the antibody reactivity, as has been previously reported for the Bw6 epitope (35). Therefore, since the SAB data of mAb DMS4G2 provides evidence that eplet 71TTS can induce antibody formation, we consider this eplet antibody-verified. The positions of the involved amino acid residues on the surface of the respective HLA molecules for all aforementioned eplets are depicted in **Figure S2**.

**Table 2:** HLA class I monoclonal antibodies tested in single antigen beads assay.

mAb	Eplet*	Reactive HLA alleles	Uniquely shared amino acids	Conclusion
**mAbs verifying eplets as included in the HLA Epitope Registry**				
JOK3H4 (IgM)	107W	A*02:01, A*02:02, A*02:03, A*02:05, A*69:01	107W	Verifies eplet 107W
OK2F3 (IgM)	161D	A*03:01	161D	Verifies eplet 161D
VTM4D9 (IgG)	65QIA (65A 66I 69A)	B*07:02, B*27:03, B*27:05, B*27:08, B*42:01, B*54:01, B*55:01, B*56:01, B*67:01, B*73:01, B*81:01, B*82:02	65Q + 66I + 69A	Verifies eplet 65QIA (65Q + 66I + 69A)
GK31F12 (IgM)	144QL (144Q 145L)	B*13:02	145L	Verifies eplet 144QL (144Q + 145L)
MUL6D1 (IgM)	151AHA (150A 151H 152A)	A*11:01, A*11:02	150A + 151H + 152A	Verifies eplet 151AHA (150A + 151H + 152A)
GV2D5 (IgG)	163RG (163R 167G)	A*01:01	163R + 166D + 167G	Verifies eplet 163RG (163R + 167G)
VP5G3 (IgM)	163RW (163R 167W)	A*11:01, A*11:02, A*25:01, A*26:01, A*43:01, A*66:01	163R + 166E + 167W	Verifies eplet 163RW (163R + 167W)
IND3H3 (IgG)	65GK (65G 66K)	A*23:01, A*24:02, A*24:03	65G	Verifies eplet 65GK (65G + 66K)
DK1G8 (IgG)	62LQ (62L 63Q)	A*29:01, A*29:02, A*43:01	62L or 63Q	Verifies eplet 62LQ (62L + 63Q)
VN2F1 (IgM)	62GRN (62G 65R 66N)	B*57:01, B*58:01	62G + 65R + 66N	Verifies eplet 62GRN (62G + 65R + 66N)
SN607D8 (IgG)	144TKH (142T 144K 145H)	A*02:01, A*02:02, A*02:03, A*02:05, A*68:01, A*68:02, A*69:01	142T or 145H	Verifies eplet 144TKH (142T + 144K + 145H)
DMS4G2 (IgG)	71TTS (71T 73T 77S)	B*07:03, B*08:01, B*14:01, B*14:02, B*15:01, B*15:02, B*15:03, B*15:12, B*15:18, B*18:01, B*35:01, B*35:08, B*38:01, B*39:01, B*40:01, B*40:02, B*41:01, B*44:02, B*44:03, B*45:01, B*48:01, B*50:01, B*78:01	71T + 73T + 77S	Verifies eplet 71TTS (71T + 73T + 77S)
**mAbs not verifying eplets as included in the HLA Epitope Registry**				
VIE6C10 (IgG)	65GK (65G 66K)	A*23:01	Inconclusive	Does not verify eplet 65GK
SN66E3 (IgM)	144TKH (142T 144K 145H)	A*02:01, A*02:02, A*02:05, A*68:01, A*68:02, A*69:01	145H + 149A *or* 144K + 145H + 149A *or* 142T + 149A *or* 142T + 145H + 149A	Does not verify eplet 144TKH, but verifies eplet 145KHA (144K + 145H + 149A)
VDK1D12 (IgM)	44KM (44K 45M [149A 150V 151H 152A 158V])	A*01:01, A*36:01	44K *or* 150V *or* 158V	Does not verify eplet 44KM. Propose to define as reactivity pattern: 44K/150V/158V
**mAbs not listed in the HLA Epitope Registry**				
DK7C11 (IgG)	n/a	B*15:12, B*44:02, B*44:03, B*45:01, B*82:02	167S and 163L + 167G	Verifies eplet 163LS/G

*Eplet definition as recorded in the HLA Epitope Registry. n/a, not applicable.

### Several Human mAbs Do Not Verify HLA Class I Eplets as Listed in the HLA Epitope Registry

**Figure 2:**
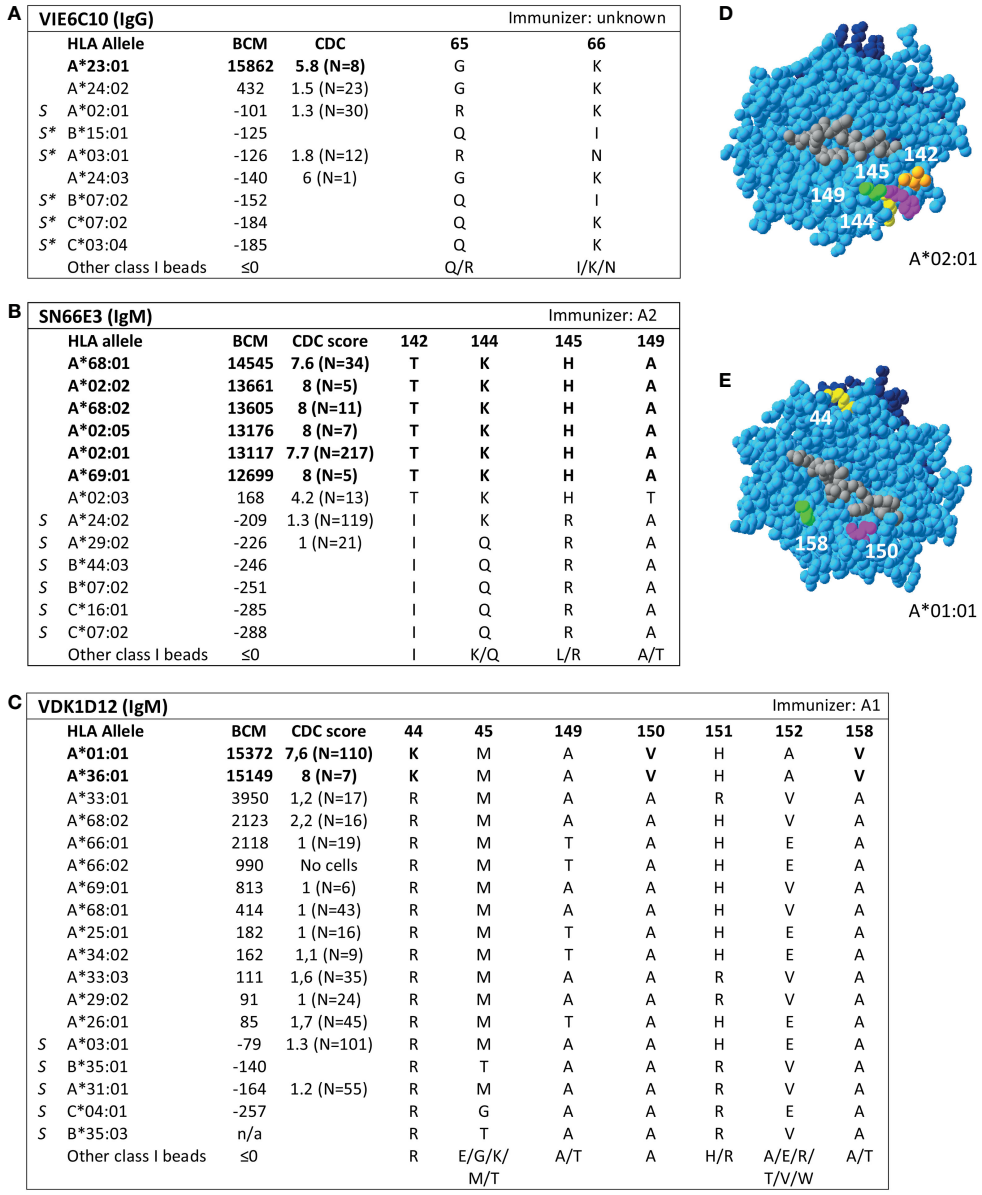
Reactivity analysis of HLA class I specific-monoclonal antibodies (mAb) that do not confirm eplets as defined in the HLA Epitope Registry. Comparison of the amino acid positions of interest of a selection of HLA class I alleles in the single antigen bead assay and complement dependent cytotoxicity assay for **(A)** mAb VIE6C10, **(B)** SN66E3 and **(C)** VDK1D12. Allele B*35:03 is a self-allele that is not present in the single antigen beads assay panel and has only 1 amino acid mismatch on position 116 with the other self-allele B*35:01. **(D)** Location of amino acids 142T (orange), 144K (yellow), 145H (magenta) and 149A (green) on the crystal structure of A*02:01 (PBD: 3UTQ). **(E)** Location of amino acids 44K (yellow), 150V (magenta) and 158V (green) on the crystal structure of A*01:01 (PBD: 3BO8). The α chain is depicted in light blue, the β chain in dark blue, and the peptide in grey. mAb concentrations used for testing were 10 µg/ml. Self HLA alleles of the antibody producer marked with * are the most likely high resolution HLA typing due to ambiguous second-field typing. Alleles in bold are considered positive. Amino acid residues in bold are uniquely shared by the reactive alleles, or are part of a uniquely shared combination of residues. BCM, background corrected mean fluorescence intensity; CDC, complement dependent cytotoxicity; PBD, Protein Data Bank; S, self HLA alleles of antibody producer.

Analysis of SAB data of three HLA class I mAbs did not verify the eplets which they were attributed to by the HLA Epitope Registry. Firstly, according to the Registry, mAb VIE6C10 verifies eplet 65GK. However, SAB and CDC results demonstrated that mAb VIE6C10 is negative for allele A*24:02, which bears eplet 65GK (**Figure 2A**). These data were confirmed in the One Lambda SAB assay (data not shown). Allele A*24:03, which also bears eplet 65GK, was also negative in SAB, but a previous CDC result showed positivity (N=1). Therefore, mAb VIE6C10 was also tested in a lower concentration of 1 ug/ml in SAB (1:10 dilution), to rule out the prozone effect, which can occur when high-titer antibodies interfere with the detection of IgG in the SAB assay (36). However, this SAB assay yielded similar results (data not shown). Reactivity analysis did not identify any other uniquely shared residue or eplet, and HLAMatchmaker analysis of the SAB data did not identify any eplet either. We therefore conclude that mAb VIE6C10 does not verify eplet 65GK.

Additionally, although mAb SN66E3 is listed in the Registry as one of two mAbs that verifies eplet 144TKH, reactivity analysis did not verify this eplet. Although the 144TKH-bearing allele HLA-A*02:03 is weakly positive in CDC (some cells bearing this allele being completely negative and some being positive), it was negative in SAB analysis. Considering A*02:03 non-reactive, the combinations of 145H + 149A *or* 142T + 149A are uniquely shared by the reactive alleles (**Figure 2B**). All three involved residues are within 3.5 Å (**Figure 2D**) and correspond to eplet 145KHA (144K 145H 149A), which was also identified by HLAMatchmaker analysis. Similarly to the analysis of mAb SN607D8, it is not possible to determine whether the combination of two or three amino acid residues is crucial for antibody induction. Consequently, we consider mAb SN66E3 as evidence for the antibody verification of eplet 145KHA.

Lastly, SAB analysis of mAb VDK1D12 yielded three uniquely shared residues; 44K, 150V and 158V (**Figure 2C**), which are not within a 3.5 Å or 15 Å distance and therefore cannot form an eplet or structural epitope (**Figure 2E**). This mAb is listed as evidence for verification of eplet 44KM, which does not fit the eplet definition as the residues (44K 45M [149A 150V 151H 152A] [158V]) exceed the 3.5 Å radius. We therefore conclude that 44KM is not an antibody-verified eplet but propose to consider 44K/150V/158V as an antibody-verified reactivity pattern (see **Box 1**).

### Reactivity Analysis of an Unlisted HLA Class I mAb

**Figure 3:**
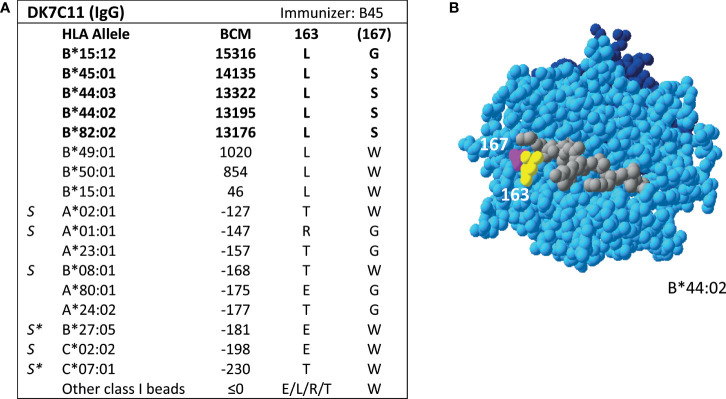
Reactivity analysis of mAb DK7C11. **(A)** Comparison of the amino acid positions of interest of a selection of HLA class I alleles in the single antigen bead assay. Monoclonal antibody concentration used for testing was 10 µg/ml. Amino acid positions in brackets are not solvent-accessible according to HLA-EMMA. Self HLA alleles of the antibody producer marked with * are the most likely high resolution HLA typing due to ambiguous second-field typing. **(B)** Location of amino acids 163L (yellow) and 1671 (magenta) on the crystal structure of B*44:02 (PBD: 1M6O). Alleles in bold are considered positive. Amino acid residues in bold are part of the combination of residues that is uniquely shared by the reactive alleles. BCM, background corrected mean fluorescence intensity; S, self HLA alleles of antibody producer; PBD, Protein Data Bank.

Additionally, SAB analysis of mAb DK7C11, which is not included in the Registry, verified eplet 163LS/G (**Figure 3**). Tested at a concentration of 1 µg/ml, three alleles with 46 ≤ MFI ≤ 1010 became negative (MFI ≤ 0), while the five positive alleles remained positive with MFIs > 4000 (data not shown). The immunizing antigen for this mAb was HLA-B45, which bears the uniquely shared residue 167S. Interestingly, the mAb showed cross-reactivity with HLA-B*15:12, for which the combination of 163L + 167G is uniquely shared. No CDC data on cells bearing HLA-B*15:12 were available. Based on these new mAb data, the level of evidence for antibody verification of eplet 163LS/G is raised from level B (patient sera) to level A1.

### Reactivity Analysis of HLA Class II-Specific Human mAbs

**Figure 4:**
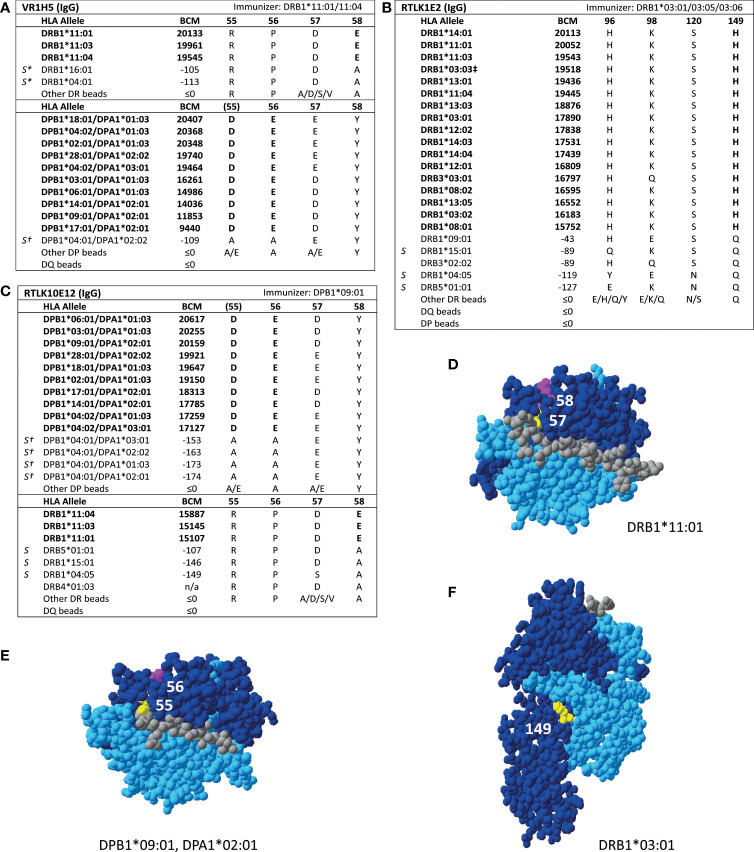
Reactivity analysis of HLA class II specific-monoclonal antibodies. Comparison of the amino acid positions of interest of a selection of DRB1 and DPB1 alleles in the single antigen bead assay for **(A)** mAb VR1H5, **(B)** RTLK10E12 and **(C)** RTLK1E2rec-IgG1 (Kramer et al. HLA. 2019 Nov;94(5):415-424.). Monoclonal antibody concentrations used for testing were 10, 2.5 and 10 µg/ml for mAb VR1H5, RTLK10E12 and RTLK1E2 respectively. Self HLA alleles of the antibody producer marked with * are the most likely high resolution HLA typing due to ambiguous second-field typing. Self-allele DRB4*01:03 for mAb RTLK10E12 is not present in the single antigen beads assay panel. **(D)** Location of amino acids 57D (yellow) and 58E (magenta) on the crystal structure of DRB1*11:01 (PBD: 6PCL). **(E)** Location of amino acids 55D (yellow) and 56E (magenta) on the crystal structure of DPA1*02:01/DPB1*09:01 (Modelled PBD: 3WEX). **(F)** Location of amino acid 149H (yellow) on the crystal structure of DRB1*03:01 (PBD: 1A6A). ^†^DPA1 typing of antibody producer is not known. ^‡^The sequence of DRB1*03:03 is not fully known. For the unknown sections (residue positions 1-5 and 95-226), the same sequence as DRB1*03:01 is assumed (C. Heylen, Immucor, personal communication, August 4, 2020). Alleles in bold are considered positive. Amino acid residues in bold are uniquely shared by the reactive alleles. BCM, background corrected mean fluorescence intensity; S, self HLA alleles of antibody producer; PBD, Protein Data Bank.

For HLA class II, four mAbs that were included in the HLA Epitope Registry without having been published and one additional mAb were tested in SAB assays (**Table 3**). HLA-DPB-specific mAb TL3B6 verified eplet 84DEAV (**Figures S3A, B**) and HLA-DRB-specific mAb BVK3D6 verified eplet 74R (**Figures S3C, D**). The HLA-DR11 induced mAb VR1H5, which was included in the HLA Epitope Registry as evidence for verification of eplet 57DE on HLA-DR and eplet 56E on HLA-DPB indeed verified eplet 57DE and was cross-reactive with eplet 56E (**Figure 4A**). Interestingly, mAb RTLK10E12, which is currently not included in the Registry, showed the same reactivity as VR1H5, but was induced by immunizing allele DPB1*09:01 and was cross-reactive with HLA-DR11 (**Figure 4B**). Hence, analysis of mAbs VR1H5 and RTLK10E12 confirm that both eplets can induce a cross-reactive antibody response and both eplets are therefore considered antibody-verified. SAB analysis of mAb RTLK1E2 was performed with the previously generated recombinant mAb RTLK1E2rec-IgG1 (37). Although RTLK1E2 is listed in the HLA Epitope Registry as evidence for antibody verification of eplet 96HK, SAB analysis showed that this mAb is reactive with allele DRB3*03:01, which does not bear eplet 96HK (**Figure 4C**). Instead, residue 149H was identified as the uniquely shared residue for mAb RTLK1E2. Since this result was not in line with the data in the HLA Epitope Registry, the mAb was also tested using One Lambda SAB assay. In concordance with our data, this assay demonstrated allele DRB3*03:01 to be reactive as well, and reactivity analysis demonstrated 149H to be uniquely shared (data not shown). Furthermore, the same reactive alleles have previously been described to be positive in a C3d SAB assay (37). Hence, based on analysis of mAb RTLK1E2, eplet 96HK cannot be regarded as antibody-verified. Instead, RTLK1E2 verifies eplet 149H, which was already present in the HLA Epitope Registry, but had not been antibody-verified yet. Localizations of eplet 57DE, 56E and 149H on the surface of HLA molecules are visualized in **Figures 4D–F**.

**Table 3:** HLA class II monoclonal antibodies tested in single antigen beads assay.

mAb	Eplet*	Reactive HLA alleles	Uniquely shared amino acids	Conclusion
**mAbs verifying eplets as included in the HLA Epitope Registry**				
TL3B6 (IgG)	84DEAV (84D 85E 86A 87V)	DPB1*01:01,DPB1*03:01, DPB1*05:01, DPB1*06:01, DPB1*09:01, DPB1*11:01, DPB1*13:01, DPB1*14:01, DPB1*17:01, DPB1*19:01	84D or 85E or 86A or 87V	Verifies eplet 84DEAV (84D + 85E + 86A + 87V)
BVK3D6 (IgM)	74R (70Q 73G 74R)	DRB1*03:01, DRB1*03:02, DRB1*03:03, DRB3*01:01	74R	Verifies eplet 74R (70Q + 73G + 74R)
VR1H5 (IgG)	DRB: 57DE (57D 58E) & DPB: 56E (55D 56E)	DRB1*11:01, DRB1*11:03, DRB1*11:04, DPB1*02:01, DPB1*03:01, DPB1*04:02, DPB1*06:01, DPB1*09:01, DPB1*14:01, DPB1*17:01, DPB1*18:01, DPB1*28:01	DRB: 58E DPB: 55D or 56E	Verifies DRB eplet 57DE (57D + 58E)
**mAbs not verifying eplets as included in the HLA Epitope Registry**				
RTLK1E2 (IgG)	96HK (96H 98K 120S)	DRB1*03:01, DRB1*03:02, DRB1*03:03, DRB1*08:01, DRB1*08:02, DRB1*11:01, DRB1*11:03, DRB1*11:04, DRB1*12:01, DRB1*12:02, DRB1*13:01, DRB1*13:03, DRB1*13:05, DRB1*14:01, DRB1*14:03, DRB1*14:04, DRB3*03:01	149H	Does not verify eplet 98HK but verifies eplet 149H
**mAbs not listed in the HLA Epitope Registry**				
RTLK10E12 (IgG)	n/a	DRB1*11:01, DRB1*11:03, DRB1*11:04, DPB1*02:01, DPB1*03:01, DPB1*04:02, DPB1*06:01, DPB1*09:01, DPB1*14:01, DPB1*17:01, DPB1*18:01, DPB1*28:01	DRB: 58E DPB: 55D or 56E	Verifies DPB eplet 56E (55D + 56E)

*Eplet definition as recorded in the HLA Epitope Registry. n/a, not applicable.

### Critical Review of All Evidence for Antibody Verification Status of Eplets in the HLA Epitope Registry

The HLA Epitope Registry databases include a total of 492 eplets of which 72 HLA class I, 36 HLA-DRB, 27 HLA-DQ and 11 HLA-DP have the antibody-verified status. In order to assign a level of evidence for antibody verification status of these eplets, a total of 121 literature references that are incorporated in the Registry were critically reviewed according to the classification in **Table 1**. Eplets with level A1 or A2 evidence were considered as truly antibody-verified, while level B, C and D were considered as provisionally antibody-verified (**Table 4**). The complete overview of all reviewed literature and level of evidence classification per eplet can be found in **Supplementary Tables 2** and **3** for HLA class I and II respectively.

**Table 4:** Classification of level of evidence for antibody-verification of HLA class I and class II eplets.

	HLA Class I	HLA-DR	HLA-DQ	HLA-DP
Antibody-verified				
A1 (human mAb included in registry)	21	4		2
A1 (new human mAb)	1	3	2	
A2 (adsorption and elution studies)	22	1	5	
B (patient sera; HLA-DP only)				2
* Total antibody-verified*	44	8	7	4
Provisionally antibody-verified				
B (patient sera)	15	18	10	6
C (low resolution HLA-typing)	1	4		
D (murine or other species mAb)	6	5		1
* Total provisionally antibody-verified*	22	27	10	7
Not antibody-verified				
According to Registry	152	84	56	51
Human mAb is peptide-dependent		1		
Eplet located in the peptide-binding groove		1		
Eplet included as ‘‘eplet pair’’ only	4	1		
* Total not antibody-verified*	156	87	56	51
Antibody-verified reactivity patterns				
A1 (human mAb included in registry)	1	1	1	
A1 (new human mAb)			3	
A2 (adsorption and elution studies)	1		6	
* Total antibody-verified reactivity patterns*	2	1	10	
Total	224	123	83	62

For HLA class I, 44 eplets were considered truly antibody-verified based on human mAb data (n=22), including the mAb data presented in this paper, and adsorption and elution studies (n=22). A number of 22 eplets were considered provisionally antibody-verified based on reactivity analysis of patient sera (n=15), CDC with serologically typed cells only (n=1) and murine mAbs (n=6). The HLA Epitope Registry included four eplets that were listed as ‘eplet pairs’. Eplet pairs were considered not antibody-verified since they consist of two separate eplets that are located too far from each other to form a single eplet (> 3.5 Å), and thus are not jointly capable of inducing an antibody response. For two eplets, 44KM and 193PL, the residues that comprise these eplets as defined by the HLA Epitope Registry exceed the 3.5 Å range (**Figures 2E** and **S4A**). Since eplet 44KM is verified by human mAb analysis and verification of eplet 193PL is based on adsorption and elution studies, we consider them as antibody-verified *reactivity patterns* (see **Box 1**), of which the actual eplets remain unknown. The overall list of HLA class I antibody-verified eplets and reactivity patterns including literature references (24, 38–42) is depicted in **Table 5**.

**Table 5:** HLA class I antibody-verified eplets and reactivity patterns.

	Polymorphic residue	Highest level of Evidence	Reference	Comment
**Eplets**				
21H	21H	A2	(38)	
41T	41T	A1	(39)	
56R	56R	A2	(38)	
62GE	62G 63E	A1	(40)^†^	
62GRN	62G 65R 66N	A1	*	SAB analysis of human mAb VN2F1 verifies eplet 62GRN (**Figure 1B**).
62LQ	62L 63Q	A1	*	SAB analysis of human mAb DK1G8 verifies eplet 62LQ (**Figure 1A**).
65GK	65G 66K	A1	*	SAB analysis of human mAb IND3H3 verifies eplet 65GK (**Figure S1H**).
65QIA	65Q 66I 69A	A1	*	SAB analysis of human mAb VTM4D9 verifies eplet 65QIA (**Figure S1C**).
69AA	69A 71A	A2	(41)	
69TNT	69T 70N 71T	A2	(41)	
70IAQ	66I 69A 70Q	A2	(41)	
71TTS	71T 73T 77S	A1	*	SAB analysis of human mAb DMS4G2 verifies eplet 71TTS (**Figure 1D**).
73TVS	73T 76V 77S	A2	(42)	
76ANT	76A 77N 80T	A2	(41)	
76ESN	76E 77S 80N	A2	(41)	
76VRN	76V 79R 80N	A2	(38)	
80I	80I	A1	(39)	
80K	80K	A2	(38)	
80N	80N	A1	(39)	
80TLR	80T 82L 83R	A2	(41)	
82LR	82L 83R	A1	(39, 40)^†^	
90D	90D	A2	(41)	
107W	107W	A1	*	SAB analysis of human mAb JOK3H4 verifies eplet 107W (**Figure S1A**).
127K	127K	A2	(41)	
144K	144K	A2	(41)	
144KR	144K 145R	A1	(40)^†^	
144QL	144Q 145L	A1	*	SAB analysis of human mAb GK31F12 verifies eplet 144QL (**Figure S1D**).
144TKH	142T 144K 145H	A1	*	SAB analysis of human mAb SN607D8 verifies eplet 144TKH (**Figure 1C**).
145KHA	144K 145H 149A	A1	(24)*	SAB analysis of human mAb SN66E3 (**Figure 2B**) verifies eplet 145KHA.
149TAH	149T 150A 151H	A2	(38)	
151AHA	150A 151H 152A	A1	*	SAB analysis of human mAb MUL6D1 verifies eplet 151AHA (**Figure S1E**).
161D	161D	A1	*	SAB analysis of human mAb OK2F3 verifies eplet 161D (**Figure S1B**).
163EW	163E 167W	A2	(41)	
163LS/G	163L 167G/S	A1	*	SAB analysis of human mAb DK7C11 verifies eplet 163L 167G/S (**Figure 3**).
163LW	163L 167W	A1	(39, 40)^†^	
163R	163R	A2	(41)	
163RG	163R 167G	A1	*	SAB analysis of human mAb GV2D5 verifies eplet 163RG (**Figure S1F**).
163RW	163R 167W	A1	*	SAB analysis of human mAb VP5G3 verifies eplet 163RW (**Figure S1G**).
166DG	166D 167G	A1	(40)^†^	
177KT	177K 178T	A2	(38)	
180E	180E	A2	(41)	
219W	219W	A1	(40)^†^	
253Q	253Q	A2	(38)	
267QE	267Q 268E	A2	(38)	
**Reactivity patterns**				
44KM	44K 45M (149A 150V1 51H 152A) (158V)	A1	*	Proposed reactivity pattern definition: 44K/150V/158V, based on SAB analysis of human mAb VDK1D12 (**Figures 2C, E**).
193PL	193P 194L (273S)	A2	(38)	Proposed reactivity pattern definition: 193P+194L/273S (**Figure S4A**).

*Evidence for antibody-verification by human mAb single antigen beads analysis is provided in this paper.

^†^This literature reference is not included yet in the HLA Epitope Registry for this eplet.

For HLA class II, we observed that for several eplets, especially HLA-DQ, the residues that comprise the eplet as defined by the HLA Epitope Registry exceed the 3.5 Å radius. For these eplets, previously published SAB data were re-analyzed to determine the uniquely shared residues and subsequently the possible eplets, which led to proposed new definitions of these reactivity patterns. The list of HLA class II antibody-verified eplets and antibody-verified reactivity patterns including literature references (43–50) are depicted in **Tables 6** and **7** respectively.

**Table 6:** HLA class II antibody-verified eplets.

Antigen	Eplet	Polymorphic residue	Highest level of Evidence	Reference	Comment
DRB	16Y	16Y 25R	A1	(43)	
DRB	25Q	25Q 30L 14K	A1	(44)^†^	Residue 30L is not within 3.5 Å distance of 14K and 25Q and is not solvent-accessible according to HLA-EMMA. Proposed new definition: 14K+25Q, based on Kramer et al.
DRB	57DE	57D 58E	A1	*	SAB analysis of human mAb VR1H5 verifies eplet 57DE (**Figure 4A**).
DRB	74R	70Q 73G 74R	A1	*	Reactivity pattern analysis of human mAb BVK3D6 verifies eplet 74R (**Figure S3C**).
DRB	77T	77T	A2	(45)	
DQB	45EV	45E 46V 47Y	A1	(46)^†^	
DQB	45GV	45G 46V	A2	(47)	
DQB	55PP	55P 56P	A2	(47)	
DQB	55R	55R	A1	(46)^†^	
DQB	77R	75V 77R	A2	(47)	
DQB	77T	77T	A2	(47)	
DQB	125SQ	125S 126Q	A2	(47)	
DPB	56A	56A	B	(48)	
DPB	56E	55D 56E	A1	*	SAB analysis of human mAb RTLK10E12 verifies eplet 56E (**Figure 4B**).
DPB	84DEAV	84D 85E 86A 87V	A1	*	SAB analysis of human mAb TL3B6 verifies eplet 84DEAV (**Figure S3A**).
DPB	85GPM	85G 86P 87M	B^‡^	(48)	
**New antibody-verified eplets**					
DRB	31FY	31F 32Y	A1	(44)^†^	Proposed new definition: 31F+32Y+37Y, based on data from on Kramer et al.
DRB	70QA	70Q 73A	A1	(44)^†^	
DRB	149H	149H	A1	*	SAB analysis of human mAb RTLK1E2 verifies eplet 149H (**Figure 4C**).

*Evidence for antibody-verification by human mAb single antigen beads analysis is provided in this paper.

^†^This literature reference is not included yet in the HLA Epitope Registry for this eplet.

^‡^Human recombinant mAb LB_DP4_A provides A1 evidence (Kramer et al. Manuscript in preparation).

**Table 7:** HLA class II antibody-verified reactivity patterns.

Antigen	Eplet	Polymorphic residue	Highest level of Evidence	Reference	Proposed new definition	Comment
DRB	98ES	98E 120S	A1	(44)^†^	78V/96H+98E/98E+120S	Residue 78V is also uniquely shared by the reactive HLA alleles (data from Kramer et al.) However, residue 78V is not within a 3.5 Å radius from the other residues (**Figure S4B**).
DQB	46VY	46V 52P 28T	A2	(47, 49)	28T/46V/52P	These 3 residues are all uniquely shared but are not within 3.5 Å (**Figure S4C**). Residue 28T is not solvent-accessible according to HLA-EMMA.
DQB	52LL	52L 55L 28S 30S 37I	A1	(46)^†^	46E/52L/55L/ 71K/74A	Not only residue 28S, 30S, 37I, 52L and 55L, but also 46E, 71K and 74 are uniquely shared by DQB1*02:01 and DQB1*02:02. These residues are not within a 3.5 Å radius (**Figure S4D**). Residues 28S, 30S and 37I are not solvent-accessible according to HLA-EMMA.
DQB	52PQ	53Q 89G 90I	A2	(47)	53Q/84E/85V/89G/90I/220R/221Q	Not only residues 53Q, 89G and 90I, but also 84E, 85V, 220R and 221Q are uniquely shared by DQB1*05 and DQB1*06. However, these residues are not within a 3.5 Å radius (**Figure S4E**).
DQB	74S	74S 26G	A1	(50)	74S/26G	Both residues are uniquely shared, but are not within a 3.5 Å radius (**Figure S4F**). Residue 26G is not solvent accessible according to HLA-EMMA.
DQB	84QL	84Q 86E 87L 89T 90T 125A	A1	(46)^†^	53L/84Q/85L/86E/87L/89T/90T/125A/220H/221H	Not only residues 84Q, 86E, 87L, 89T, 90T and 125A but also 53L, 220H and 221 are uniquely shared by DQB1*02, DQB1*03 and DQB1*04. However, these residues are not within a 3.5 Å radius (**Figure S4G**).
DQB	116I	116I 125S	A2	(47)	116I/125S/224R	Residues 116I, 125S and 224R are all uniquely shared but not within a 3.5 Å radius (**Figure S4H**). 116I is not solvent accessible according to HLA-EMMA.
DQB	182N	182N	A1	(46)^†^	52P+53L/140T/182N	Not only residue 182N, but also 52P+53L and 140T are uniquely shared by DQB1*03 and DQB1*04, but are not within a 3.5 Å radius (**Figure S4I**).
DQB	182S	182S	A2	(47)	140A/182S	Not only residue 182S, but also 140A is uniquely shared by DQB1*02, DQB1*05 and DQB1*06. However, the residues are not within a 3.5 Å radius (**Figure S4J**).
DQA	40GR	40G 47C 50V 51L	A2	(47, 49)	40G/47C/50V/51L/53Q	Not only residues 40G, 47C, 50V and 51L, but also 53Q is uniquely shared by DQA1*04, DQA1*05 and DQA1*06. However, the residues are not within a 3.5 Å radius (**Figure S4K**.
DQA	47KHL	47K 52H 54L	A2	(47)	47K/52H/54L	These 3 residues are all uniquely shared but are not within a 3.5 Å radius (**Figure S4L**).

^†^This literature reference is not included yet in the HLA Epitope Registry for this eplet.

For HLA-DR, five eplets were considered antibody-verified based on human mAb data that were included in the Registry (n=4) and based on reactivity analysis of adsorbed and eluted antibodies (n=1). Three previously not-verified eplets could be verified based on recent literature that was not yet included in the Registry (eplets 31FY and 70QA) (44) and based on new human mAb analysis in this current paper (eplet 149H). 27 eplets were considered provisionally antibody-verified based on reactivity analysis of patient sera (n=18), CDC with low resolution HLA typed cells only (n=4) and murine mAbs (n=5). Three eplets listed as antibody-verified by the HLA Epitope Registry were considered not antibody-verified. Eplet 11STS was not considered antibody-verified because the amino acids defining this eplet are located on the bottom of the peptide-binding groove (45), making it very unlikely that it is accessible for the B cell receptor and can induce antibody formation. Eplet 67LQ was not considered antibody-verified as it was solely listed as an eplet pair, and eplet 30C was considered not antibody-verified because binding of the mAb that was used for verification is peptide-dependent (51, 52) and no other evidence was available.

The residues defining eplet 98ES exceed the 3.5 Å distance, which is therefore considered as an antibody-verified reactivity pattern (**Figure S4B**). Eplet 96HK is provisionally antibody-verified (level B) as human mAb data analysis showed that not eplet 96HK but eplet 149H was uniquely shared (**Figure 4C**), leaving patient sera tested in SAB assay (published on the HLA Epitope Registry website, not peer-reviewed) as highest level of evidence for eplet 96HK. Furthermore, we propose to redefine eplet 25Q (25Q 30L 14K) to 14K + 25Q, since residue 30L is not solvent accessible and is not a within 3.5 Å radius of residues 14K and 25Q (44).

For HLA-DQ, 10 of the antibody-verified eplets exceed the 3.5 Å radius and are therefore considered as antibody-verified reactivity patterns (**Figures S4C–L**). We consider seven eplets truly antibody-verified based on new mAb data (n=2) and adsorption and elution experiments (n=5). The remaining 10 eplets are provisionally antibody-verified based on patient sera.

For HLA-DP, two eplets were antibody-verified based on human mAb data and seven eplets were provisionally antibody-verified based on reactivity analysis of patient sera (n=6) and a murine mAb (n=1). An exception regarding antibody verification classification was made for eplets 56A and 85GPM, of which the highest level of evidence is patient sera. These eplets were considered antibody-verified because of the extensive analysis on multiple sera performed by Cano et al. (48), and the fact that these particular HLA-DP epitopes are well established (53). Additionally, unpublished data from our own laboratory provides A1 evidence for eplet 85GPM (Kramer et al. manuscript in preparation).

Overall, we consider 44 HLA class I eplets and 19 HLA class II eplets as being truly antibody-verified and a total of two HLA class I and 11 HLA class II reactivity patterns as being antibody-verified.

## Discussion

The HLA Epitope Registry and HLAMatchmaker have formed the foundation for the vast majority of clinical studies investigating the role of HLA eplets in transplantation. In this study, we have critically reviewed the evidence for the antibody verification status of eplets included in the HLA Epitope Registry. The different methodologies that are currently used for antibody-verification do not represent the same level of evidence for the antibody-verified status of eplets. However, while previously a category of ‘provisionally verified’ was present, the current dichotomous yes or no antibody-verified status in the HLA Epitope Registry does not take the heterogeneity in the level of evidence into account. To provide insight on what basis an eplet is considered antibody-verified by the Registry, we have introduced a classification system to score the level of evidence. Our results show that for many eplets, especially for HLA class II, the antibody-verified status is based on sera from multi- or uni-parous women or transplant patients, experiments with only serologically typed cells, or murine mAbs. However, we argue that these methods are not suitable for definitive antibody verification of eplets. Although SAB analysis of sera from immunized individuals can be informative, the reactivity of sera tested in SAB is in most, if not all cases the result of a polyclonal antibody response. These patterns are often broad and do not permit the identification of a single HLA eplet, since the pattern of reactive HLA alleles is caused by multiple antibodies recognizing several HLA epitopes. Even seemingly narrow SAB reactivity may be caused by more than one eplet mismatch. For several other eplets, antibody verification status was based on experiments using serologically typed cells only. These cells are not suitable for state-of-the-art reactivity analysis due to the low resolution of HLA typing, which makes definitive assignment of the inducing eplet very difficult. Furthermore, for 11 eplets only reactivity analysis of murine mAbs was available. Murine mAbs are generated by immunization with HLA but do not necessarily recognize the same epitopes as human mAbs, since immunogenicity of HLA antigens is affected by the recipients’ HLA type (22). Therefore, we argue that if antibody-verified status in the HLA Epitope Registry is solely based on reactivity analysis of patient sera, experiments with serologically typed cells or murine mAbs, this should result in provisional evidence for antibody verification, but not a definitive antibody-verified status. In the first report of the antibody verification of eplets in the HLA Epitope Registry, antibody-verified eplets were classified as ‘confirmed’ or ‘provisional’ depending on the amount and degree of evidence that was available (18). However, this classification was removed in the second update of the Registry (23).

Aside from eplets, the HLA Epitope Registry also includes ‘‘eplet pairs’’, of which a number have been assigned the antibody-verified status. HLA eplets are based on the concept that one or multiple mismatched amino acid residues induce the humoral immune response through interaction with the CDR3 region of the B cell receptor heavy chain. Accordingly, the residues that constitute an eplet should be in a 3.5 Å radius (4). However, eplet pairs consist of two eplets (a combination of a nonself-eplet and a self-eplet) that are located within the 15 Å radius that constitutes the structural epitope, but are not within 3.5 Å from each other (54). Therefore, eplet pairs cannot be regarded as the configuration that induces the antibody response and subsequently, we did not consider eplet pairs for antibody verification.

Our review of the HLA Epitope Registry does not only provide insight in the heterogeneity of the level of evidence of eplet antibody verification, but also demonstrates that a substantial portion of the presented mAb data and patient sera analyses had not been published in peer-reviewed journals. Aiming to substantiate the antibody-verified status of eplets based on human mAbs which reactivity analyses have not been published previously, we tested these mAbs in SAB assays and performed reactivity analysis. For the majority of mAbs tested, the identified uniquely shared amino acids indeed corresponded with the eplet. However, SAB analysis of three mAbs did not confirm the antibody verification of the eplet as assigned by the Registry. The analyses of mAbs SN66E3 and RTLK1E2 supported the verification of two different eplets, while no inducing eplet could be determined for mAb VIE6C10. Furthermore, the reactivity analyses of SN607D8 and SN66E3 identified multiple uniquely shared residues or uniquely shared combinations of two residues, while the corresponding eplets, 144TKH and 145KHA respectively, are defined by three residues. Based on our analyses, it is possible that not all three, but only one or two residues are crucial for the induction of anti-HLA antibodies. For these eplets, this difference in possible eplet definitions is clinically relevant, since there are less common, but intermediately and well-documented alleles that bear only one of the uniquely shared residues (55). Consequently, using the definition that includes all three residues could possibly disregard patients with these less common HLA alleles in respect to HLA eplet matching purposes. Mutation studies of HLA alleles or testing of the mAbs against a panel of cells containing these less common HLA types could provide more insight in the actual configuration of polymorphic residues that comprises these eplets. However currently, experimental possibilities are limited due to the lack of suitable reagents.

Detailed analysis of the localization of antibody-verified eplet configurations on crystalized HLA structures demonstrated that not all antibody-verified eplets in the HLA Epitope Registry comply with the eplet definition. Especially for HLA-DQ, the polymorphic residues that comprise the eplet configuration are often too distant (> 3.5 Å) from each other to form an eplet. Re-analysis of previously published SAB data of human mAbs and eluted antibodies from patient sera demonstrated that for 10 HLA-DQ, one HLA-DR and two HLA class I eplets multiple uniquely shared amino acids could be identified that were not within 3.5 Å. Because these residues are simultaneously present on the Common HLA alleles in the CIWD 3.0.0 (55) with only a few exceptions, we propose to consider these configurations as antibody-verified reactivity patterns instead of eplets. Accordingly, these antibody-verified reactivity patterns can still be considered in HLA matching strategies and molecular mismatch evaluation for the vast majority of transplant patients. For four reactivity patterns there is a small number of Common HLA alleles that can be considered as an exception, which are listed in **Supplementary Table 4**. For instance, the antibody-verified reactivity pattern 74S/26G is present on all Common DQB1*04 and DQB1*05 alleles. There are also two alleles that bear 26G, but have 74E instead of 74S (DQB1*03:05 and DQB1*03:25). When a patient carrying DQB1*03:01 (which lacks this reactivity pattern) would receive a transplant from a DQB1*03:05 donor, only one of the two residues of this reactivity pattern would be mismatched, namely 26G. At this moment it is not clear whether residue 26G or residue 74S is crucial for antibody induction and therefore it is uncertain whether the 26G mismatch in this case would be clinically relevant. Structural data based on mutation studies and crystallography are required to determine the true binding place of the antibody to be able to determine which of the residues of a reactivity pattern can be considered as the true eplet.

The rationale for this explicit and precise definition of eplets and reactivity patterns also follows from the need to define the most immunogenic eplets in transplantation (56). Multiple studies have tried to identify the most immunogenic eplet mismatches (16, 57–59), which is a crucial step in making eplet-matching in transplantation clinically applicable. Currently, these studies are limited by the use of different versions of the HLA Epitope Registry and/or HLAMatchmaker and consequently the different eplet definitions that are used in the analyses. For example, a recent paper investigating the immunogenicity of HLA-DQ eplets used HLAMatchmaker 2.1 to determine eplet mismatches (59). In this version of HLAMatchmaker, eplets 84QL and 125A are considered as separate eplets. In the current version of the HLA Epitope Registry however, eplet 125A has been removed. In fact, residue 125A has been added to the definition of eplet 84QL, since residues 84Q, 86E, 87L, 89T, 90T and are all uniquely shared by alleles DQB1*02, DQB1*03 and DQB1*04, but are not within 3.5 Å. Hence, according to our proposed classification, eplet 84QL rather is a reactivity pattern. Accordingly, the use of different definitions for the same eplet and the inclusion of eplet pairs in immunogenicity studies distorts the interpretation and comparability of immunogenicity scores. Furthermore, inconsistencies in eplet definition and antibody-verified status between the HLA Epitope Registry and HLAMatchmaker (60) and the lack of documentation of previous versions of the Registry hamper investigations towards eplet mismatch loads and transplant outcomes.

The SAB assays in this study were performed with the Lifecodes SAB assay from Immucor. It has been demonstrated that the beads of the other manufacturer of these assays (One Lambda, Thermofisher) are bound with an admixture of intact and denatured HLA (61, 62), while the Immucor assay predominantly contains intact HLA (63). The presence of denatured HLA on beads can results in detection of antibodies against cryptic epitopes (64). Since these antibodies will not bind to intact HLA, cellular testing of mAbs or eluted antibodies can exclude the possibility of an antibody directed towards a cryptic epitope. The clinical relevance of antibodies against cryptic epitopes in transplantation remains questionable and warrants further investigation.

This critical review of the antibody-verified status of eplets in the HLA Epitope Registry has demonstrated that the level of evidence of antibody-verified eplets is heterogeneous and that not all data have been published in peer-reviewed journals. Analysis of luminex SAB data of human mAbs showed that not all mAbs verified the eplets they were assigned to. Since an increasing number of clinical studies investigate eplet mismatch load as a risk factor for inferior transplant outcomes and seek to identify the most immunogenic eplet mismatches, it is vital to define a set of well-defined antibody-verified eplets in a transparent manner. Our list of antibody-verified eplets and reactivity patterns is the first step towards a uniform and transparent method of eplet definition and antibody verification. However, eplets that are considered provisionally verified, or not antibody-verified could still play a clinically relevant role in transplantation, since antibody verification is limited by the available reagents and patient material. In this respect it is important for our field to collaborate in the yet uncompleted endeavor of eplet antibody verification. For future publications, we propose to set a standard of required data regarding antibody verification that should be published. Preferably, this includes reactivity analysis of human mAb data tested in SAB assay or antibodies eluted from immunized patient sera. The used SAB panel and HLA typing of the antibody producer and immunizer should also be included to make re-analysis and thorough interpretation of the data possible. Finally, we propose to establish an international committee that oversees nomenclature and antibody verification of eplets to facilitate the establishment of a well-documented, transparent list of eplets with antibody verification status classified by the level of evidence, striving for better comparable results in clinical and immunogenicity studies on the road to eplet matching in transplantation.

## Data Availability Statement

The raw data supporting the conclusions of this article will be made available by the authors, without undue reservation.

## Author Contributions

SB reviewed the literature and analyzed the data. KB performed laboratory experiments. SB and SH wrote the manuscript. CK, JF, MR, AM, and FC critically reviewed the manuscript. All authors contributed to the article and approved the final version of the manuscript.

## Conflict of Interest

The authors declare that the research was conducted in the absence of any commercial or financial relationships that could be construed as a potential conflict of interest.

## Publisher’s Note

All claims expressed in this article are solely those of the authors and do not necessarily represent those of their affiliated organizations, or those of the publisher, the editors and the reviewers. Any product that may be evaluated in this article, or claim that may be made by its manufacturer, is not guaranteed or endorsed by the publisher.

## Abbreviations

Å, Ångstrom; CDC, complement-dependent cytotoxicity; HLA, human leukocyte antigen; HLA-EMMA, HLA Epitope Mismatch Algorithm; mAb, monoclonal antibody; MFI, mean fluorescence intensity; SAB, single antigen beads.
