# Supplementary material for: A Comprehensive Evaluation of the Antibody-Verified Status of Eplets Listed in the HLA Epitope Registry
Source: Front Immunol. 2022 Jan 28;12:800946. doi: 10.3389/fimmu.2021.800946 (PMC8831796; doi:10.3389/fimmu.2021.800946)

## Supplementary Figures

**Figure S1**: Reactivity analysis of HLA class I specific-monoclonal antibodies that confirm eplets as defined in the HLA Epitope Registry…………………………………………………………………………………………………………………………………………… 1

**Figure S2**: Amino acid positions of antibody-verified HLA class I eplets on the surface of HLA molecules.................. 2

**Figure S3**: Reactivity analysis of HLA class II specific-monoclonal antibodies that confirm eplets as defined in the HLA Epitope Registry…………………………………………………………………………………………………………………………………………… 4

**Figure S4:** Amino acid positions of antibody-verified HLA class I, HLA-DR and HLA-DQ reactivity patterns on the surface of HLA molecules…………………………………………………………………………………………………………………………………… 5

**Figure S1.** Reactivity analysis of HLA class I specific-monoclonal antibodies that confirm eplets as defined in the HLA Epitope Registry. Comparison of the amino acid positions of interest of a selection of HLA class I alleles in the single antigen bead assay for mAb JOK3H4 (A), OK2F3 (B), VTM4D9 (C), GK31F12 (D) MULD6D1 (E), GV2D5 (F), VP5G3 (G) and IND3H3 (H). Monoclonal antibody concentrations used for testing were 10 µg/ml. Amino acid positions in brackets are not solvent-accessible according to HLA-EMMA. Self HLA alleles of the antibody producer marked with * are the most likely high resolution HLA typing due to ambiguous second-field typing. Self HLA alleles marked with † are the most likely high resolution typing converted from serological typing. Alleles in bold are considered positive. Amino acid residues in bold are part of the combination of residues that is uniquely shared by the reactive alleles.
BCM, background corrected mean fluorescence intensity; S, self HLA alleles of antibody producer

| A | **JOK3H4 (IgM)** | | Immunizer: unknown |
|---|---|---|---|
| | **HLA allele** | **BCM** | **107** |
| | **A*02:01** | **2263** | **W** |
| | **A*02:05** | **2116** | **W** |
| | **A*69:01** | **2043** | **W** |
| | **A*02:02** | **1919** | **W** |
| | **A*02:03** | **1782** | **W** |
| *S* | B*08:01 | -179 | G |
| *S* | A*01:01 | -200 | G |
| *S* | A*24:02 | -202 | G |
| *S* | C*04:01 | -250 | G |
| *S* | C*07:01 | -271 | G |
| *S* | B*35:02 | n/a | G |
| | Other class I beads | ≤0 | G |

| B | **OK2F3 (IgM)** | | Immunizer: A3 |
|---|---|---|---|
| | **HLA allele** | **BCM** | **161** |
| | **A*03:01** | **12333** | **D** |
| *S* | A*02:01 | -146 | E |
| *S** | B*27:05 | -169 | E |
| *S* | A*68:01 | -191 | E |
| *S* | B*07:02 | -226 | E |
| *S* | C*02:02 | -227 | E |
| *S** | C*07:02 | -249 | E |
| | Other class I beads | ≤0 | E |

| C | **VTM4D9 (IgG)** | | | | Immunizer: B7 |
|---|---|---|---|---|---|
| | **HLA allele** | **BCM** | **65** | **66** | **69** |
| | **B*27:08** | **20886** | **Q** | **I** | **A** |
| | **B*67:01** | **20617** | **Q** | **I** | **A** |
| | **B*81:01** | **19986** | **Q** | **I** | **A** |
| | **B*82:02** | **19849** | **Q** | **I** | **A** |
| | **B*56:01** | **19788** | **Q** | **I** | **A** |
| | **B*42:01** | **19512** | **Q** | **I** | **A** |
| | **B*55:01** | **19396** | **Q** | **I** | **A** |
| | **B*07:02** | **19254** | **Q** | **I** | **A** |
| | **B*27:05** | **18634** | **Q** | **I** | **A** |
| | **B*27:03** | **15978** | **Q** | **I** | **A** |
| | **B*73:01** | **13817** | **Q** | **I** | **A** |
| | **B*54:01** | **10296** | **Q** | **I** | **A** |
| | C*03:04 | 544 | Q | K | R |
| | B*15:16 | 532 | R | N | A |
| | B*46:01 | 505 | Q | K | R |
| | C*03:03 | 99 | Q | K | R |
| | C*01:02 | 78 | Q | K | R |
| *S* | B*15:01 | -109 | Q | I | T |
| *S* | A*25:01 | -112 | R | N | A |
| *S* | B*44:03 | -177 | Q | I | T |
| *S* | A*29:02 | -193 | R | N | A |
| *S* | C*05:01 | -212 | Q | K | R |
| *S* | C*16:01 | -218 | Q | K | R |
| | Other class I beads | ≤0 | G/Q/R | I/K/N | A/R/T |

| D | **GK31F12 (IgM)** | | | Immunizer: B13 |
|---|---|---|---|---|
| | **HLA allele** | **BCM** | **144** | **145** |
| | **B*13:02** | **11864** | **Q** | **L** |
| *S* | A*02:01 | -133 | K | H |
| *S* | A*03:01 | -159 | K | R |
| *S* | B*15:01 | -180 | Q | R |
| *S* | B*35:01 | -195 | Q | R |
| *S* | C*03:04 | -249 | Q | R |
| *S* | C*04:01 | -261 | Q | R |
| | Other class I beads | ≤0 | K/Q | H/R |

| E | **MUL6D1 (IgM)** | | | | Immunizer: A*11:01 |
|---|---|---|---|---|---|
| | **HLA allele** | **BCM** | **150** | **151** | **152** |
| | **A*11:01** | **10523** | **A** | **H** | **A** |
| | **A*11:02** | **10490** | **A** | **H** | **A** |
| *S* | A*25:01 | -169 | A | H | E |
| | A*01:01 | -209 | V | H | A |
| *S* | B*18:01 | -214 | A | R | V |
| | A*36:01 | -218 | V | H | A |
| *S* | B*51:01 | -233 | A | R | E |
| *S* | C*15:02 | -258 | A | R | E |
| | C*07:01 | -289 | A | R | A |
| | C*16:01 | -295 | A | R | A |
| | C*07:02 | -307 | A | R | A |
| *S* | A*02:06 | n/a | A | H | V |
| *S* | C*12:03 | n/a | A | R | E |
| | Other class I beads | ≤0 | A | H/R | E/R/T/V/W |

| F | **GV2D5 (IgG)** | | | | Immunizer: A1 |
|---|---|---|---|---|---|
| | **HLA allele** | **BCM** | **163** | **166** | **(167)** |
| | **A*01:01** | **13713** | **R** | **D** | G |
| | B*59:01 | 18 | T | E | W |
| | A*11:01 | 11 | R | E | W |
| | B*37:01 | 5 | T | E | W |
| | A*23:01 | -8 | T | D | G |
| *S†* | A*29:01 | -26 | T | E | W |
| *S†* | B*57:01 | -28 | L | E | W |
| *S†* | A*02:01 | -38 | T | E | W |
| *S†* | B*44:02 | -49 | L | E | S |
| | B*15:12 | -50 | L | D | G |
| | A*80:01 | -64 | E | D | G |
| | A*24:02 | -67 | T | D | G |
| *S†* | C*06:02 | -88 | T | E | W |
| | Other class I beads | ≤0 | E/L/R/T | E | S/W |

| G | **VP5G3 (IgM)** | | | | Immunizer: A26 |
|---|---|---|---|---|---|
| | **HLA allele** | **BCM** | **163** | **166** | **(167)** |
| | **A*66:01** | **15087** | **R** | **E** | **W** |
| | **A*25:01** | **14948** | **R** | **E** | **W** |
| | **A*26:01** | **12554** | **R** | **E** | **W** |
| | **A*11:02** | **12193** | **R** | **E** | **W** |
| | **A*11:01** | **12085** | **R** | **E** | **W** |
| | **A*43:01** | **9535** | **R** | **E** | **W** |
| *S** | B*40:01 | -195 | E | E | W |
| | A*01:01 | -201 | R | D | G |
| *S** | A*32:01 | -211 | T | E | W |
| *S** | B*18:01 | -211 | T | E | W |
| *S** | A*24:02 | -226 | T | D | G |
| *S** | C*03:04 | -282 | L | E | W |
| *S** | C*07:01 | -301 | T | E | W |
| | Other class I beads | ≤0 | E/L/T | D/E | S/G/W |

| H | **IND3H3 (IgG)** | | | Immunizer: A23 |
|---|---|---|---|---|
| | **HLA allele** | **BCM** | **65** | **66** |
| | **A*24:02** | **13052** | **G** | **K** |
| | **A*23:01** | **12323** | **G** | **K** |
| | **A*24:03** | **11429** | **G** | **K** |
| *S* | A*32:01 | -73 | R | N |
| *S** | A*01:01 | -95 | R | N |
| *S** | C*07:01 | -106 | Q | N |
| *S** | B*08:01 | -111 | Q | I |
| *S** | B*51:01 | -117 | Q | I |
| *S* | C*01:02 | -143 | Q | K |
| | Other class I beads | ≤0 | Q/R | I/K/N |

**Figure S2.** Amino acid positions of HLA class I eplets on the surface of HLA molecules. (A) Location of amino acid 107W (yellow) on the crystal structure of A*02:01 (PBD: 3UTQ). (B) Location of amino acids 150A (yellow), 151H (magenta) and 152A (green) on the crystal structure of A*11:01 (PBD: 1X7Q). (C) Location of amino acid 161D (yellow) on the crystal structure of A*03:01 (PBD: 3RL1). (D) Location of amino acids 163R (yellow), 166D (magenta) and 167G (green) on the crystal structure of A*01:01 (PBD: 3BO8). (E) Location of amino acids 65Q (yellow), 66I (magenta) and 69A (green) on the crystal structure of B*07:02 (PBD: 4U1H). (F) Location of amino acids 163R (yellow), 166E (magenta) and 167W (green) on the crystal structure of A*11:01 (PBD: 1X7Q). (G) Location of amino acids 144Q (yellow) and 145L (magenta) on the crystal structure of B*44:02 (PBD: 1M6O) (H) Location of amino acids 65G (yellow) and 66K (magenta) on the crystal structure of A*24:01 (PBD: 3WL9). (I) Location of amino acids 62L (yellow) and 63Q (magenta) on the crystal structure of A*03:01 (PBD: 3RL1). (J) Location of amino acids 62G (yellow), 65R (magenta) and 66N (green) on the crystal structure of B*58:01 (PBD: 5IND). (K) Location of amino acids 142T (yellow), 144K (magenta) and 145H (green) on the crystal structure of A*02:01 (PBD: 3UTQ). (L) Location of amino acids 71T (yellow), 73T (magenta) and 77S (green) on the crystal structure of B*15:01 (PBD: 1XR9). The α chain is depicted in light blue, the β chain in dark blue, and the peptide in grey.
PBD, Protein Data Bank

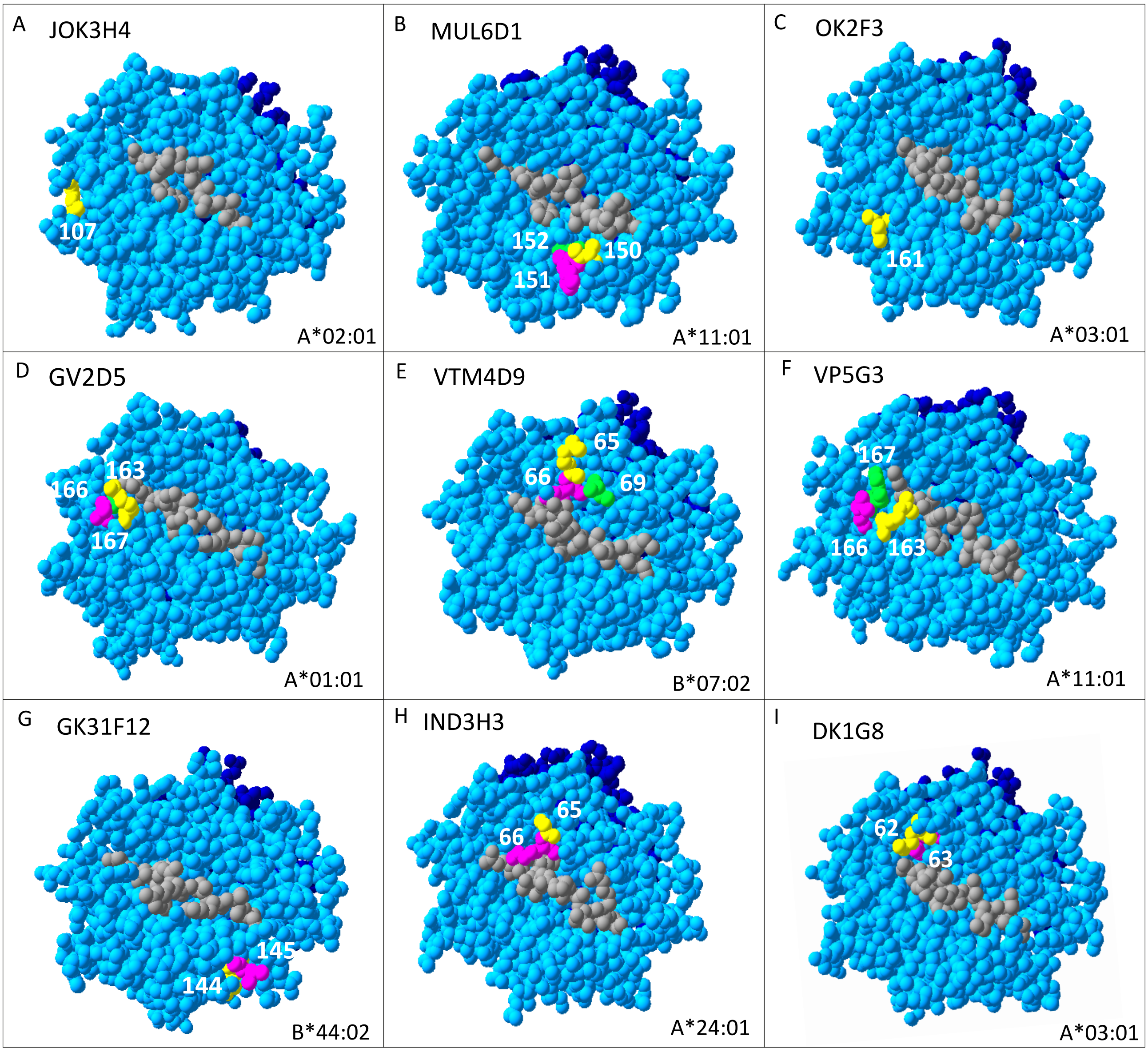


**Figure continues on the next page**

**Figure S2 continued.**

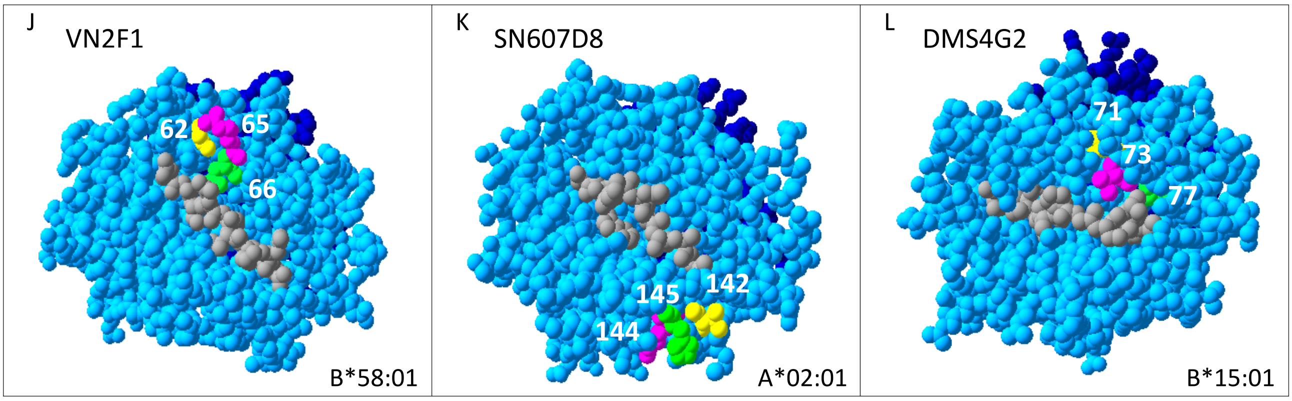

**Figure S3.** Reactivity analysis of HLA class II specific-monoclonal antibodies that confirm eplets as defined in the HLA Epitope Registry. (A) Comparison of the amino acid positions of interest of a selection of DPB1 alleles in the single antigen bead assay for mAb TL3B6. Monoclonal antibody concentration used for testing was 10 µg/ml. (B) Location of amino acids 84E (yellow), 85E (magenta), 86A (green) and 87V (orange) on the crystal structure of DPA1*02:02/DPB1*05:01 (Modelled PBD: 3WEX). The α chain is depicted in light blue, the β chain in dark blue, and the peptide in grey. (C) Comparison of the amino acid positions of interest of a selection of DRB1 alleles in the single antigen bead assay for mAb BVK3D6. Monoclonal antibody concentration used for testing was 4 µg/ml. Self HLA alleles of the antibody producer marked with * are the most likely high resolution HLA typing converted from serological typing. Self-allele DRB3*02:01 is not present in the single antigen beads assay panel. (D) Location of amino acids 70Q (yellow), 73G (magenta) and 74 (green) on the crystal structure of DRB1*03:01 (PBD: 1A6A). The α chain is depicted in light blue, the β chain in dark blue, and the peptide in grey. Alleles in bold are considered positive. Amino acid residues in **bold** are uniquely shared by the reactive alleles. BCM, background corrected mean fluorescence intensity; S, self HLA alleles of antibody producer; PBD, Protein Data Bank

A

| **TL3B6 (IgG)** | | | | | | Immunizer: DPB1*13:01 |
|---|---|---|---|---|---|---|
| | **HLA Allele** | **BCM** | **84** | **85** | **86** | **87** |
| | **DPB1*03:01/DPA1*01:03** | **18387** | **D** | **E** | **A** | **V** |
| | **DPB1*05:01/DPA1*02:02** | **17717** | **D** | **E** | **A** | **V** |
| | **DPB1*19:01/DPA1*02:01** | **17450** | **D** | **E** | **A** | **V** |
| | **DPB1*13:01/DPA1*04:01** | **17373** | **D** | **E** | **A** | **V** |
| | **DPB1*05:01/DPA1*02:01** | **17295** | **D** | **E** | **A** | **V** |
| | **DPB1*06:01/DPA1*01:03** | **17063** | **D** | **E** | **A** | **V** |
| | **DPB1*01:01/DPA1*02:02** | **16851** | **D** | **E** | **A** | **V** |
| | **DPB1*14:01/DPA1*02:01** | **16694** | **D** | **E** | **A** | **V** |
| | **DPB1*09:01/DPA1*02:01** | **16520** | **D** | **E** | **A** | **V** |
| | **DPB1*05:01/DPA1*03:01** | **16481** | **D** | **E** | **A** | **V** |
| | **DPB1*01:01/DPA1*03:01** | **16380** | **D** | **E** | **A** | **V** |
| | **DPB1*01:01/DPA1*01:03** | **16374** | **D** | **E** | **A** | **V** |
| | **DPB1*13:01/DPA1*02:01** | **16224** | **D** | **E** | **A** | **V** |
| | **DPB1*01:01/DPA1*02:01** | **16098** | **D** | **E** | **A** | **V** |
| | **DPB1*11:01/DPA1*02:01** | **15785** | **D** | **E** | **A** | **V** |
| | **DPB1*17:01/DPA1*02:01** | **15320** | **D** | **E** | **A** | **V** |
| *S* | DPB1*04:01/DPA1*02:02 | -85 | G | G | P | M |
| *S* | DPB1*02:01/DPA1*01:03 | -178 | G | G | P | M |
| | Other DP beads | ≤0 | G/V | G | P | M |
| | DR beads | ≤0 | | | | |
| | DQ beads | ≤0 | | | | |

B

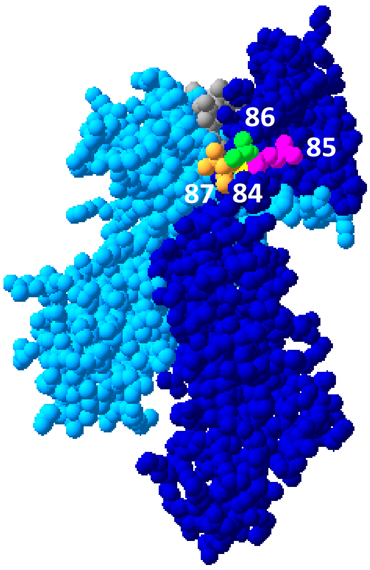


DPB1*05:01, DPA1*02:02

C

| **BVK3D6 (IgM)** | | | | | Immunizer: unknown |
|---|---|---|---|---|---|
| | **HLA Allele** | **BCM** | **70** | **73** | **74** |
| | **DRB1*03:03** | **13342** | Q | G | **R** |
| | **DRB3*01:01** | **13273** | Q | G | **R** |
| | **DRB1*03:01** | **13025** | Q | G | **R** |
| | **DRB1*03:02** | **11747** | Q | G | **R** |
| | DRB3*02:02 | 518 | Q | G | Q |
| | DRB3*03:01 | 200 | Q | G | Q |
| *S** | DRB1*11:01 | -105 | D | A | A |
| *S** | DRB1*15:01 | -122 | Q | A | A |
| *S** | DRB5*01:01 | -153 | D | A | A |
| *S** | DRB3*02:01 | n/a | Q | G | Q |
| | Other DR beads | ≤0 | D/Q/R | A/G | A/E/L/Q |
| | DQ beads | ≤0 | | | |
| | DP beads | ≤0 | | | |

D

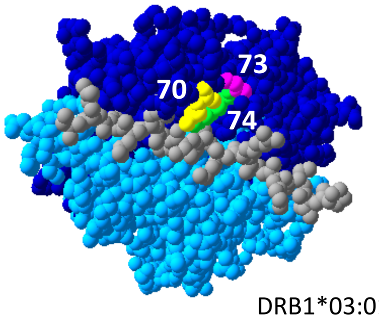


DRB1*03:01

**Figure S4.** Amino acid positions of antibody-verified HLA class I, HLA-DR and HLA-DQ reactivity patterns on the surface of HLA molecules. (A) Location of amino acids 193P (yellow), 194L (magenta) and 273S of reactivity pattern 193PL on the crystal structure of C*07:02 (modelled PBD: 4NT6). (B) Location of amino acid 78V (yellow), 96H (magenta), 98E (green) and 120S (orange) of reactivity pattern 98ES on the crystal structure of DRB1*07:01 (modelled PBD: 4I5B). (C) Location of amino acid 46V (yellow) and 52P (magenta) of reactivity pattern 46VY on the crystal structure of DQB1*06:02/ DQA1*01:02 (PBD: 1UVQ). (D) Location of amino acids 46E (yellow), 52L (magenta), 55L (green), 71K (orange) and 74A (red) of reactivity pattern 52LL on the crystal structure of DQB1*02:01/DQA1*05:01 (PBD: 1S9V). (E) Location of amino acid 53Q (yellow), 84E (magenta), 85V (green), 89G (orange) and 90I (red) of reactivity pattern 52PQ on the crystal structure of DQB1*06:02/DQA1*01:02 (PBD: 1UVQ) The position of residues 220R and 221Q could not be determined, as these amino acids are not included in the crystal structure. (F) Location of amino acids 26G (yellow) and 74S (magenta) of reactivity pattern 74S on the crystal structure of DQB1*05:01/DQA1*01:02 (modelled PBD: 3PL6). (G) Location of amino acids 53L (yellow), 84Q (magenta), 85L (orange), 86E (green), 87L (red), 89T (pink), 90T (yellow) and 125A (yellow) of reactivity pattern 84QL on the crystal structure of DQB1*03:02/DQA-1*03:01 (PBD: 4Z7U). The position of residues 220H and 221H could not be determined, as these amino acids are not included in the crystal structure. (H) Location of amino acids 116I (yellow) and 125S (magenta) of reactivity pattern 116I on the crystal structure of DQB1*05:01/DQA1*01:02 (modelled PBD: 3PL6). The position of residue 224R could not be determined, as these amino acids are not included in the crystal structure. (I) Location of amino acids 52P (yellow), 53L (magenta), 140T (green) and 182N (orange) of reactivity pattern 182N on the crystal structure of DQB1*03:02/DQA1*03:01 (PBD: 4Z7U). (J) Location of amino acids 140A (yellow) and 182S (magenta) of reactivity pattern 182S on the crystal structure of DQB1*06:02/DQA1*01:02 (PBD: 1UVQ). (K) Location of amino acids 40G (yellow), 47C (magenta), 50V (green), 51L (orange) and 53Q (red) of reactivity pattern 40GR on the crystal structure of DQB1*02:02/DQA1*05:01 (PBD: 1S9V). (L) Location of amino acids 47K (yellow), 52H (magenta) and 54L (green) of reactivity pattern 47KHL on the crystal structure of DQB1*03:03/DQA1*02:01 (PBD: 4Z7U). The α chain is depicted in light blue, the β chain in dark blue, and the peptide in grey.
PBD, Protein Data Bank

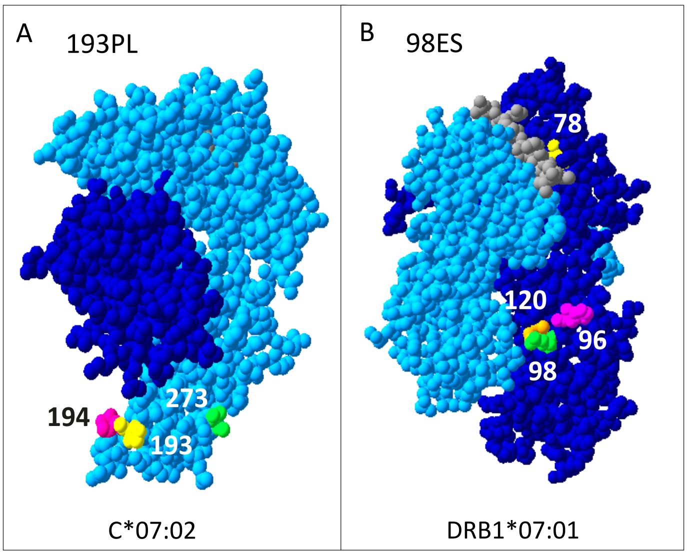


**Figure continues on the next page**

**Figure S4 continued.**

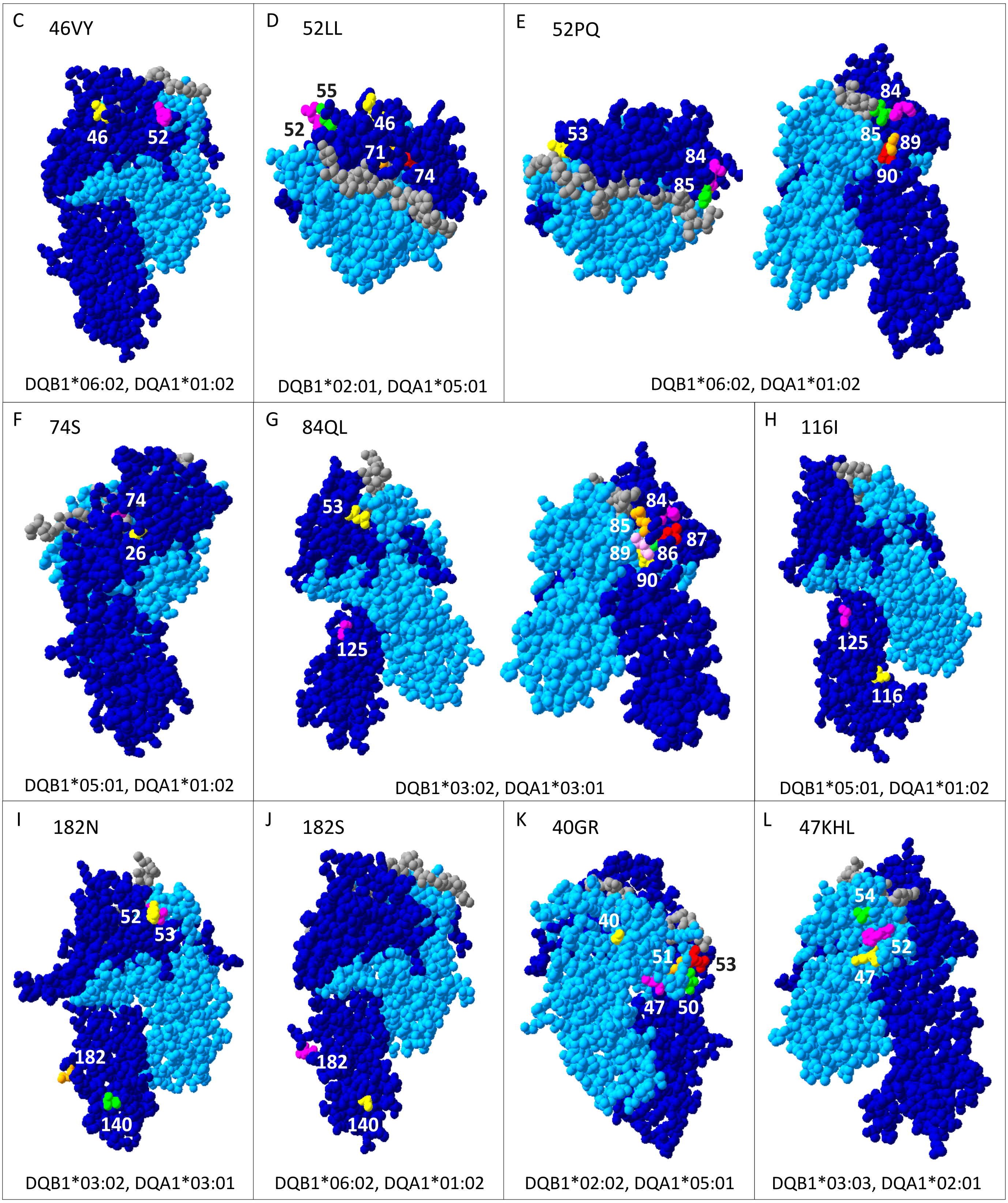

Supplement: Supplementary file 1 [file DataSheet_1.pdf]
