# Supplementary material for: A Comprehensive Evaluation of the Antibody-Verified Status of Eplets Listed in the HLA Epitope Registry
Source: Front Immunol. 2022 Jan 28;12:800946. doi: 10.3389/fimmu.2021.800946 (PMC8831796; doi:10.3389/fimmu.2021.800946)
Supplement: Supplementary file 2 [file DataSheet_2.pdf]

Supplementary Tables

Supplementary Table 1. Single antigen beads assay panel that was used for analysis……………………………2

Supplementary Table 2. Complete overview of all HLA class I eplets that are considered antibody-verified by the HLA Epitope Registry………………………………………………………………………………………………3

Supplementary Table 3. Complete overview of all HLA class II eplets that are considered antibody-verified by the HLA Epitope Registry……………………………………………………………………………………………….10

Supplementary Table 4. Residue differences in reactivity patterns on Common HLA alleles………………..20

References……21

Supplementary Table 1. Single antigen beads assay panel that was used for analysis[1]

HLA class I			HLA class II		
A*01:01	B*08:01	B*51:01	DRB1*01:01	DRB3*01:01	DQA1*05:01/DQB1*03:01
A*02:01	B*13:02	B*52:01	DRB1*01:02	DRB3*02:02	DQA1*05:01/DQB1*04:01
A*02:02	B*14:01	B*53:01	DRB1*01:03	DRB3*03:01	DQA1*06:01/DQB1*03:01
A*02:03	B*14:02	B*54:01	DRB1*03:01	DRB4*01:01	DQA1*06:01/DQB1*03:03
A*02:05	B*15:01	B*55:01	DRB1*03:02	DRB5*01:01	DQA1*06:01/DQB1*04:02
A*03:01	B*15:02	B*56:01	DRB1*03:03	DRB5*02:02	DPA1*01:03/DPB1*01:01
A*11:01	B*15:03	B*57:01	DRB1*04:01	DQA1*01:01/DQB1*05:01	DPA1*01:03/DPB1*02:01
A*11:02	B*15:12	B*58:01	DRB1*04:02	DQA1*01:02/DQB1*05:01	DPA1*01:03/DPB1*03:01
A*23:01	B*15:13	B*59:01	DRB1*04:03	DQA1*01:02/DQB1*05:02	DPA1*01:03/DPB1*04:01
A*24:02	B*15:16	B*67:01	DRB1*04:04	DQA1*01:02/DQB1*06:02	DPA1*01:03/DPB1*04:02
A*24:03	B*15:18	B*73:01	DRB1*04:05	DQA1*01:02/DQB1*06:04	DPA1*01:03/DPB1*06:01
A*25:01	B*18:01	B*78:01	DRB1*07:01	DQA1*01:03/DQB1*06:01	DPA1*01:03/DPB1*18:01
A*26:01	B*27:03	B*81:01	DRB1*08:01	DQA1*01:03/DQB1*06:03	DPA1*02:01/DPB1*01:01
A*29:01	B*27:05	B*82:02	DRB1*08:02	DQA1*01:04/DQB1*05:03	DPA1*02:01/DPB1*04:01
A*29:02	B*27:08	C*01:02	DRB1*09:01	DQA1*01:04/DQB1*06:01	DPA1*02:01/DPB1*05:01
A*30:01	B*35:01	C*02:02	DRB1*10:01	DQA1*02:01/DQB1*02:01	DPA1*02:01/DPB1*09:01
A*31:01	B*35:08	C*03:03	DRB1*11:01	DQA1*02:01/DQB1*02:02	DPA1*02:01/DPB1*11:01
A*32:01	B*37:01	C*03:04	DRB1*11:03	DQA1*02:01/DQB1*03:02	DPA1*02:01/DPB1*13:01
A*33:01	B*38:01	C*04:01	DRB1*11:04	DQA1*02:01/DQB1*04:01	DPA1*02:01/DPB1*14:01
A*33:03	B*39:01	C*04:03	DRB1*12:01	DQA1*02:01/DQB1*06:01	DPA1*02:01/DPB1*15:01
A*34:02	B*40:01	C*05:01	DRB1*12:02	DQA1*03:01/DQB1*03:01	DPA1*02:01/DPB1*17:01
A*36:01	B*40:02	C*06:02	DRB1*13:01	DQA1*03:01/DQB1*03:02	DPA1*02:01/DPB1*19:01
A*43:01	B*41:01	C*07:01	DRB1*13:03	DQA1*03:01/DQB1*04:02	DPA1*02:02/DPB1*01:01
A*66:01	B*42:01	C*07:02	DRB1*13:05	DQA1*03:02/DQB1*02:02	DPA1*02:02/DPB1*04:01
A*66:02	B*44:02	C*08:01	DRB1*14:01	DQA1*03:02/DQB1*03:01	DPA1*02:02/DPB1*05:01
A*68:01	B*44:03	C*08:02	DRB1*14:03	DQA1*03:02/DQB1*03:02	DPA1*02:02/DPB1*28:01
A*68:02	B*45:01	C*12:02	DRB1*14:04	DQA1*03:02/DQB1*03:03	DPA1*03:01/DPB1*01:01
A*69:01	B*46:01	C*14:02	DRB1*15:01	DQA1*04:01/DQB1*03:03	DPA1*03:01/DPB1*04:01
A*74:01	B*47:01	C*15:02	DRB1*15:02	DQA1*04:01/DQB1*04:01	DPA1*03:01/DPB1*04:02
A*80:01	B*48:01	C*16:01	DRB1*15:03	DQA1*04:01/DQB1*04:02	DPA1*03:01/DPB1*05:01
B*07:02	B*49:01	C*17:01	DRB1*16:01	DQA1*05:01/DQB1*02:01	DPA1*04:01/DPB1*04:01
B*07:03	B*50:01	C*18:01	DRB1*16:02	DQA1*05:01/DQB1*02:02	DPA1*04:01/DPB1*13:01

[1]Immucor single antigen bead assay HLA class I lot 3005842, 3009007, 3009260, 3009478, 3009741, 3010277 and class II lot 3009113.

Supplementary Table 2. Complete overview of all HLA class I eplets that are considered antibody-verified by the HLA Epitope Registry

Eplet	Polymorphic residue	Reference	Level of evidence[1]	Additional remarks
21H	21H	El-Awar et al. 2007 Transplantation	A2	
41T	41T	El-Awar et al. 2007 Hum Immunol	A2	
		Duquesnoy et al. 2012 Hum Immunol	A1	
		Duquesnoy et al. 2016 Int J Immunogenet	B	
		Fernandez-Vina et al. 2006 Immunobiology of the Human MHC - Proceedings of the 13th International Histocompatibility Workshop and Conference - Volume I	A1	This publication is not peer-reviewed.
<u>44KM</u>	44K 45M (149A 150V1 51H 152A) (158V)	El-Awar et al. 2007 Hum Immunol	D	Eplet definition in this publication: 44K/150V/158V.
		Fernandez-Vina et al. 2006 Immunobiology of the Human MHC - Proceedings of the 13th International Histocompatibility Workshop and Conference - Volume I	A1	This publication is not peer-reviewed.
		Tongio et al. 1997 HLA: Genetic diversity of HLA: Functional and Medical Implications Volume 1 - Proceedings of the Twelfth International Histocompatibility Workshop	D	This publication is not peer-reviewed.
		HLA Epitope Registry website*	A1	Proposed new reactivity pattern definition: 44K/150V/158V, based on SAB analysis of human mAb VDK1D12 (Figure 2C).
44RMA	44R 45M 46A	El-Awar et al. 2010 Hum Immunol	D	Eplet definition in this publication: 46A.
		Duquesnoy et al. 2016 Int J Immunogenet	B	
44RT	44R 45T	El-Awar et al. 2007 Transplantation	D	Eplet definition in this publication: 45T.
45KE	45K 46E	Cauquil et al. 2018 Hla	B	
56R	56R	El-Awar et al. 2007 Transplantation	A2	
		El-Awar et al. 2010 Hum Immunol	D	
		Duquesnoy et al. 2016 Int J Immunogenet	X	This reference does not provide any evidence for the antibody-verification of eplet 56R.
		Tongio et al. 1997 HLA: Genetic diversity of HLA: Functional and Medical Implications Volume 1 - Proceedings of the Twelfth International Histocompatibility Workshop	D	This publication is not peer-reviewed.
		Nelson et al. 1989 Immunobiology of HLA Volume I: Histocompatibility Testing	D	This publication is not peer-reviewed.
62EE	62E 63E	El-Awar et al. 2007 Transplantation	D	Eplet definition in this publication: 62E.
		Fernandez-Vina et al. 2006 Immunobiology of the Human MHC - Proceedings of the 13th International Histocompatibility Workshop and Conference - Volume I	X	This reference does not provide evidence for the antibody-verification of eplet 62EE.
		HLA Epitope Registry website	X	Reactivity pattern analysis of human mAb BVK5C4 does not verify eplet 62EE but verifies eplet 166DG (Duquesnoy et al. 2013 Hum Immunol).
62GE	62G 63E	Mulder et al. 2005 J Immunol	X	This reference does not provide evidence for the antibody-verification of eplet 62GE.
		Duquesnoy et al. 2013 Hum Immunol	A1	This reference is not yet included in the HLA Epitope Registry for this eplet.
		El-Awar et al. 2007 Hum Immunol	A2	Eplet definition in this publication: 62G.
		Fernandez-Vina et al. 2006 Immunobiology of the Human MHC - Proceedings of the 13th International	A1	This publication is not peer-reviewed.

		Histocompatibility Workshop and Conference - Volume I		
		Tongio et al. 1997 HLA: Genetic diversity of HLA: Functional and Medical Implications Volume 1 - Proceedings of the Twelfth International Histocompatibility Workshop	D	This publication is not peer-reviewed.
62GK	62G 66K (74H 77D)	El-Awar et al. 2007 Hum Immunol	D	Possible eplet is defined as 43Q+62G/62G+66K/62G+76V/ 62G+79G.
		Fernandez-Vina et al. 2006 Immunobiology of the Human MHC - Proceedings of the 13th International Histocompatibility Workshop and Conference - Volume I	D	This publication is not peer-reviewed.
		Tongio et al. 1997 HLA: Genetic diversity of HLA: Functional and Medical Implications Volume 1 - Proceedings of the Twelfth International Histocompatibility Workshop	D	This publication is not peer-reviewed.
		Nelson et al. 1989 Immunobiology of HLA Volume I: Histocompatibility Testing	D	This publication is not peer-reviewed.
62GRN	62G 65R 66N	Mulder et al. 1997 Hum Immunol	C	
		HLA Epitope Registry website	X	The analysis of human mAb SEE5G7 is solely published on the HLA Epitope Registry website and is not peer-reviewed.
		El-Awar et al. 2007 Transplantation	D	Possible eplet is defined as 43P+62G/ 41A+43P+62G/ 17R+41A+ 43P+62G/ 19E+41A+ 43P+62G.
		Tongio et al. 1997 HLA: Genetic diversity of HLA: Functional and Medical Implications Volume 1 - Proceedings of the Twelfth International Histocompatibility Workshop	C	This publication is not peer-reviewed.
		HLA Epitope Registry website*	A1	SAB analysis of human mAb VN2F1 verifies eplet 62GRN (Figure 1B).
62LQ	62L 63Q	El-Awar et al. 2007 Hum Immunol	D	Eplet definition in this publication: 62L.
		HLA Epitope Registry website*	A1	SAB analysis of human mAb DK1G8 verifies eplet 62LQ (Figure 1A).
		Tongio et al. 1997 HLA: Genetic diversity of HLA: Functional and Medical Implications Volume 1 - Proceedings of the Twelfth International Histocompatibility Workshop	D	This publication is not peer-reviewed.
62RR	62R 65R	El-Awar et al. 2007 Transplantation	D	
65GK	65G 66K	El-Awar et al. 2007 Hum Immunol	D	Eplet definition in this publication: 65G.
		HLA Epitope Registry website*	X	SAB analysis of human mAb VIE6C10 does not confirm the antibody-verification of 65GK (Figure 2A).
		HLA Epitope Registry website*	A1	SAB analysis of human mAb IND3H3 verifies eplet 65GK (Figure S1H).
		Nelson et al. 1989 Immunobiology of HLA Volume I: Histocompatibility Testing	D	This publication is not peer-reviewed.
65QIA	65Q 66I 69A	El-Awar et al. 2007 Hum Immunol	A2	Eplet definition in this publication: 65Q+69A.
		HLA Epitope Registry website*	A1	SAB analysis of human mAb VTM4D9 verifies eplet 65QIA (Figure S1C).
		Tongio et al. 1997 HLA: Genetic diversity of HLA: Functional and Medical Implications Volume 1 - Proceedings of the Twelfth International Histocompatibility Workshop	D	This publication is not peer-reviewed.
69AA	69A 71A	El-Awar et al. 2007 Hum Immunol	A2	Eplet definition in this publication: 69A+43P.
69TNT	69T 70N 71T	Duquesnoy et al. 2016 Int J Immunogenet	B	
		El-Awar et al. 2007 Hum Immunol	A2	Eplet definition in this publication: 69T.
70IAQ	66I 69A 70Q	Duquesnoy et al. 2016 Int J Immunogenet	B	
		El-Awar et al. 2007 Hum Immunol	A2	Eplet definition in this publication: 65Q+69A+70Q.

71ATD	71A 73T 77D	El-Awar et al. 2007 Transplantation	D	Possible eplet is defined as 65Q+69A+ 80T 65Q+ 69A+82L 65Q+69A+ 83R.
		Duquesnoy et al. 2016 Int J Immunogenet	B	
71SA	70S 71A	El-Awar et al. 2007 Hum Immunol	D	Possible eplet is defined as 43P+65R/ 65R+163L/ 66N+131S/ 66N+163L.
71TTS	71T 73T 77S	HLA Epitope Registry website*	A1	SAB analysis of human mAb DMS4G2 verifies eplet 71TTS (Figure 1D).
		Fernandez-Vina et al. 2006 Immunobiology of the Human MHC - Proceedings of the 13th International Histocompatibility Workshop and Conference - Volume I	A1	This publication is not peer-reviewed.
73AN	73A 77N	HLA Epitope Registry website	B	This data is solely published on the HLA Epitope Registry website and is not peer-reviewed.
73TVS	73T 76V 77S	Akaza et al. 2006 Clin Transpl	A2	Possible eplet is defined as (73T)+76V+80N+90A in this study. This publication is not peer-reviewed.
76ANT	76A 77N 80T	Duquesnoy et al. 2016 Int J Immunogenet	B	
		El-Awar et al. 2007 Hum Immunol	A2	Eplet definition in this publication: 76A.
76EG	76E 79G	HLA Epitope Registry website	B	This data is solely published on the HLA Epitope Registry website and is not peer-reviewed.
76ESI	76E 77S 80I	HLA Epitope Registry website	B	This data is solely published on the HLA Epitope Registry website and is not peer-reviewed.
		Vilella et al. 1983 Hum Immunol	D	
		Nelson et al. 1989 Immunobiology of HLA Volume I: Histocompatibility Testing	D	This publication is not peer-reviewed
		Fernandez-Vina et al. 2006 Immunobiology of the Human MHC - Proceedings of the 13th International Histocompatibility Workshop and Conference - Volume I	D	This publication is not peer-reviewed
76ESN	76E 77S 80N	El-Awar et al. 2007 Hum Immunol	A2	Possible eplet is defined as 76E+80N/76E+82R/76E+83G.
		Lutz et al. 1994 J Immunol	D	
76VRN	76V 79R 80N	Duquesnoy et al. 2016 Int J Immunogenet	B	
		El-Awar et al. 2007 Transplantation	A2	Possible eplet is defined as 76V+80N/73T+76V+79R.
		HLA Epitope Registry website	B	This data is published on the HLA Epitope Registry website and is not peer-reviewed.
79GT	79G 80T	Duquesnoy et al. 2016 Int J Immunogenet	B	
80I	80I	El-Awar et al. 2007 Hum Immunol	A2	
		Duquesnoy et al. 2012 Hum Immunol	A1	
80K	80K	El-Awar et al. 2007 Transplantation	A2	Eplet definition in this paper: 77N+80K.
		Duquesnoy et al. 2011 Transpl Immunol	X	This reference does not provide evidence for the antibody-verification of eplet 80K.
		Bachelet et al. 2011 Am J Transplant	B	
80N	80N	El-Awar et al. 2007 Hum Immunol	A2	
		Duquesnoy et al. 2012 Hum Immunol	A1	
80TLR	80T 82L 83R	El-Awar et al. 2007 Hum Immunol	A2	Possible eplet is defined as 76E+80T/ 79R+80T/ 80T+82L/ 80T+83R.
82LR	82L 83R	El-Awar et al. 2007 Hum Immunol	A2	
		Duquesnoy et al. 2012 Hum Immunol	A1	
		Duquesnoy et al. 2013 Hum Immunol	A1	This reference is not yet included in the HLA Epitope Registry.
90D	90D	Duquesnoy et al. 2016 Int J Immunogenet	B	
		El-Awar et al. 2007 Hum Immunol	A2	
107W	107W	Mulder et al. 2005 J Immunol	C	
		HLA Epitope Registry website*	A1	SAB analysis of human mAb JOK3H4 verifies eplet 107W (Figure S1A).

		El-Awar et al. 2007 Hum Immunol	D	
		Fernandez-Vina et al. 2006 Immunobiology of the Human MHC - Proceedings of the 13th International Histocompatibility Workshop and Conference - Volume I	D	This publication is not peer-reviewed.
		Tongio et al. 1997 HLA: Genetic diversity of HLA: Functional and Medical Implications Volume 1 - Proceedings of the Twelfth International Histocompatibility Workshop	D	This publication is not peer-reviewed.
		Nelson et al. 1989 Immunobiology of HLA Volume I: Histocompatibility Testing	D	This publication is not peer-reviewed.
		Layet et al. 1987 J Immunol	D	
127K	127K	Duquesnoy et al. 2016 Int J Immunogenet	B	
		El-Awar et al. 2007 Hum Immunol	A2	
		El-Awar et al. 2010 Hum Immunol	D	
		De Vito et al. 1993 Hum Immunol	C	
		Laundy et al. 2004 Transfusion	B	
131S	131S	Duquesnoy et al. 2016 Int J Immunogenet	B	
138K	138K	Duquesnoy et al. 2011 Transpl Immunol	B	
138MI	138M 142I	Duquesnoy et al. 2016 Int J Immunogenet	B	
		HLA Epitope Registry website	B	This data is solely published on the HLA Epitope Registry website and is not peer-reviewed.
144K	144K	El-Awar et al. 2007 Hum Immunol	A2	
		HLA Epitope Registry website	B	This data is published on the HLA Epitope Registry website and is not peer-reviewed.
144KR	144K 145R	Duquesnoy et al. 2013 Hum Immunol	A1	This reference is not yet included in the HLA Epitope Registry for this eplet.
		El-Awar et al. 2007 Hum Immunol	A2	Possible eplet is defined as 142I+144K/144K+145R.
		Fernandez-Vina et al. 2006 Immunobiology of the Human MHC - Proceedings of the 13th International Histocompatibility Workshop and Conference - Volume I	A1	This publication is not peer-reviewed.
144QL	144Q 145L	HLA Epitope Registry website*	A1	SAB analysis of human mAb GK31F12 verifies eplet 144QL (Figure S1D).
		El-Awar et al. 2007 Hum Immunol	D	Possible eplet is defined as 145L/41T+46A.
		Mulder et al. 1997 Hum Immunol	C	
		Fernandez-Vina et al. 2006 Immunobiology of the Human MHC - Proceedings of the 13th International Histocompatibility Workshop and Conference - Volume I	X	This reference does not provide evidence for the antibody verification of 144QL.
		Tongio et al. 1997 HLA: Genetic diversity of HLA: Functional and Medical Implications Volume 1 - Proceedings of the Twelfth International Histocompatibility Workshop	C & D	This publication is not peer-reviewed.
		Nelson et al. 1989 Immunobiology of HLA Volume I: Histocompatibility Testing	D	This publication is not peer-reviewed.
144TKH	142T 144K 145H	HLA Epitope Registry website*	A1	SAB analysis of human mAb SN607D8 verifies eplet 144TKH. (Figure 1C).
		HLA Epitope Registry website	X	SAB analysis of human mAb SN66E3 does not confirm the antibody verification of eplet 144TKH, but verifies eplet 145KHA (Figure 2B).
		El-Awar et al. 2007 Hum Immunol	A2	Possible eplet is defined as 142T/145H.
		Fernandez-Vina et al. 2006 Immunobiology of the Human MHC - Proceedings of the 13th International	A1	This publication is not peer-reviewed.

		Histocompatibility Workshop and Conference - Volume I		
		Mulder et al. 1997 Hum Immunol	C	
		Muller et al. 1983 Hum Immunol	D	
		Dessi et al. 1990 Hum Immunol	C	
		Tongio et al. 1997 HLA: Genetic diversity of HLA: Functional and Medical Implications Volume 1 - Proceedings of the Twelfth International Histocompatibility Workshop	D	This publication is not peer-reviewed.
		Nelson et al. 1989 Immunobiology of HLA Volume I: Histocompatibility Testing	D	This publication is not peer-reviewed.
145KHA	144K 145H 149A	Tongio et al. 1997 HLA: Genetic diversity of HLA: Functional and Medical Implications Volume 1 - Proceedings of the Twelfth International Histocompatibility Workshop	D	This publication is not peer-reviewed.
		Fernandez-Vina et al. 2006 Immunobiology of the Human MHC - Proceedings of the 13th International Histocompatibility Workshop and Conference - Volume I	D	This publication is not peer-reviewed.
		Mulder et al. 1993 Tissue Antigens	A1	This eplet is also verified by SAB analysis of human mAb SN66E3 (Figure 2B).
		Hogan et al. 1988 J Immunol	D	
145RT	145R 149T	Duquesnoy et al. 2016 Int J Immunogenet	B	
		El-Awar et al. 2007 Hum Immunol	D	Possible eplet is defined as (9Y)+149T/ (74D)+149T.
149TAH	149T 150A 151H	El-Awar et al. 2007 Transplantation	A2	Eplet definition in this publication: 149T.
150AAH	149A 150A 151H	El-Awar et al. 2010 Hum Immunol	D	
151AHA	150A 151H 152A	HLA Epitope Registry website*	A1	SAB analysis of human mAb MUL6D1 verifies eplet 151AHA (Figure S1E).
		El-Awar et al. 2007 Transplantation	A2	Possible eplet is defined as 149A+150A+ 163R/ 149A+158A+ 163R/149A+163R+ 166E/ 149A+ 163R+ 167W.
156DA	156D 158A	Lomago et al. 2010 Hum Immunol	B	
158T	158T	El-Awar et al. 2007 Hum Immunol	D	
		Ge et al. 1993 Hum Immunol	C	
161D	161D	HLA Epitope Registry website*	A1	SAB analysis of human mAb OK2F3 verifies eplet 161D (Figure S1B).
		El-Awar et al. 2007 Hum Immunol	D	
		Fernandez-Vina et al. 2006 Immunobiology of the Human MHC - Proceedings of the 13th International Histocompatibility Workshop and Conference - Volume I	A1	This publication is not peer-reviewed.
		Tongio et al. 1997 HLA: Genetic diversity of HLA: Functional and Medical Implications Volume 1 - Proceedings of the Twelfth International Histocompatibility Workshop	C and D	This publication is not peer-reviewed.
		Nelson et al. 1989 Immunobiology of HLA Volume I: Histocompatibility Testing	D	This publication is not peer-reviewed.
163EW	163E 167W	El-Awar et al. 2007 Hum Immunol	A2	Possible eplet defined as 163E+166E/163E+167W.
163LS/G	163L 167G/S	HLA Epitope Registry website	B	This data is solely published on the HLA Epitope Registry website and is not peer-reviewed.
		*	A1	SAB analysis of human mAb DK7C11 verifies eplet 163L 167G/S (Figure 3).
163LW	163L 167W	Duquesnoy et al. 2012 Hum Immunol	A1	

		Duquesnoy et al. 2013 Hum Immunol	A1	This reference is not yet included in the HLA Epitope Registry for this eplet.
		El-Awar et al. 2007 Transplantation	A2	
		Mulder et al. 1997 Hum Immunol	C	
		Fernandez-Vina et al. 2006 Immunobiology of the Human MHC - Proceedings of the 13th International Histocompatibility Workshop and Conference - Volume I	A1	This publication is not peer-reviewed.
163R	163R	El-Awar et al. 2007 Hum Immunol	A2	
		Laundy et al. 2004 Transfusion	B	
163RG	163R 167G	HLA Epitope Registry website*	A1	SAB analysis of human mAb GV2D5 verifies eplet 163RG (Figure S1F).
		Fernandez-Vina et al. 2006 Immunobiology of the Human MHC - Proceedings of the 13th International Histocompatibility Workshop and Conference - Volume I	D	This publication is not peer-reviewed.
		El-Awar et al. 2009 Clin Transpl	B	Eplet definition in this publication: 158V+163R.<br>This reference is not yet included in the HLA Epitope Registry for this eplet. This publication is not peer-reviewed.
163RW	163R 167W	HLA Epitope Registry website*	A1	SAB analysis of human mAb VP5G3 verifies eplet 163RW (Figure S1G).
		Mulder et al. 1998 Tissue Antigens	C	
		El-Awar et al. 2007 Hum Immunol	A2	Possible eplet defined as 163R+166E/ 158A+163R/ 163R+167W.
		Fernandez-Vina et al. 2006 Immunobiology of the Human MHC - Proceedings of the 13th International Histocompatibility Workshop and Conference - Volume I	A1	This publication is not peer-reviewed.
166DG	166D 167G	El-Awar et al. 2007 Hum Immunol	A2	Eplet definition in this publication: 166D/167G.
		Fernandez-Vina et al. 2006 Immunobiology of the Human MHC - Proceedings of the 13th International Histocompatibility Workshop and Conference - Volume I	D	This publication is not peer-reviewed.
		HLA Epitope Registry website	B	This data is solely published on the HLA Epitope Registry website and is not peer-reviewed.
		Duquesnoy et al. 2013 Hum Immunol	A1	This reference is not yet included in the HLA Epitope Registry for this eplet.
173K	173K	Akaza et al. 2006 Clin Transpl	B	Eplet definition in this publication: 163L+173K.<br>This publication is not peer-reviewed.
		Duquesnoy et al. 2011 Transpl Immunol	B	
177KT	177K 178T	El-Awar et al. 2007 Transplantation	A2	
		Duquesnoy et al. 2011 Transpl Immunol	B	
		HLA Epitope Registry website	B	This data is solely published on the HLA Epitope Registry website and is not peer-reviewed.
180E	180E	El-Awar et al. 2007 Hum Immunol	A2	Eplet definition in this paper: 177D/180E.
		HLA Epitope Registry website	B	This data is solely published on the HLA Epitope Registry website and is not peer-reviewed.
193PL	193P 194L (273S)	El-Awar et al. 2007 Hum Immunol	A2	Eplet definition in this paper: 194L.<br>Proposed reactivity pattern definition based on re-analysis of SAB data from this publication: 193P+194L / 273S.
		Duquesnoy et al. 2011 Transpl Immunol	B	
193PV	193P 194V	Adeyi et al. 2005 Transpl Immunol	B	
219W	219W	Duquesnoy et al. 2013 Hum Immunol	A1	This reference is not yet included in the HLA Epitope Registry for this eplet.
		Fernandez-Vina et al. 2006 Immunobiology of the Human MHC - Proceedings of the 13th International	X	This reference does not provide evidence for the antibody verification of 219W. This publication is not peer-reviewed.

		Histocompatibility Workshop and Conference - Volume I†		
		El-Awar et al. 2009 Clin Transpl	B	This reference is not yet included in the HLA Epitope Registry for this eplet. This publication is not peer-reviewed.
248M	248M (6K) (99C)	HLA Epitope Registry website	B	This data is solely published on the HLA Epitope Registry website and is not peer-reviewed.
253Q	253Q	El-Awar et al. 2007 Transplantation	A2	
		HLA Epitope Registry website	B	This data is published on the HLA Epitope Registry website and is not peer-reviewed.
267QE	267Q 268E	El-Awar et al. 2007 Transplantation	A2	Eplet definition in this paper: 267Q.

[1]A1, Human monoclonal antibody + single antigen bead (SAB) assay and/or complement dependent cytotoxicity assay (CDC) with high resolution HLA typed cells; A2, Adsorption and elution studies + SAB and/or CDC with high resolution HLA typed cells; B, Patient serum tested in SAB; C, Human monoclonal antibody or adsorption and elution studies or patient sera tested with serologically typed cells; D, Other species (e.g. murine monoclonal antibody).
*Single antigen bead analysis of this human mAb is provided in this paper.
Underlined: The residues of these eplets exceed the 3.5 Ångstrom radius and we therefore propose to consider these eplets as antibody-verified reactivity patterns. See Table 7 for proposed definitions and rationale.

Supplementary Table 3. Complete overview of all HLA class II eplets that are considered antibody-verified by the HLA Epitope Registry

Antigen	Eplet	Polymorphic residue	Reference	Level of evidence[1]	Additional remarks
DRB	4Q	4Q	HLA Epitope Registry website	B	This data is solely published on the HLA Epitope Registry website and is not peer-reviewed.
			Knowles et al. 1986 J Immunol	D	
			Knowles 1989 Immunobiology of HLA Volume I: Histocompatibility Testing	D	This publication is not peer-reviewed.
			Lardy et al. 1991 Hum Immunol	D	
			Fu et al. 1992 Hum Immunol	D	
			Cai et al. 2006 Clin Transpl	B	This publication is not peer-reviewed.
			Cai et al. 2006 Am J Transplant	B	
DRB	4R	4R	Marsh et al. 1989 Immunol Today	D	
			Ogasawara et al. 1985 Tissue Antigens	D	
			Fu et al. 1992 Hum Immunol	D	
			Fu et al. 1994 Hum Immunol	D	
DRB	11STS	9E 10Y 11S 12T 13S	Cai et al. 2006 Am J Transplant	X	This publication defines the potential eplets as 11S/12T/13S using adsorption and elution of alloantibodies from patient sera. This eplet is considered not antibody-verified because it is found on the bottom of the peptide-binding groove and therefore cannot induce antibody-formation.
			Heyes et al. 1992 HLA 1991 - Proceedings of the Eleventh International Histocompatibility Workshop and Conference – Volume 2	X	This publication includes murine mAb analysis and is not peer-reviewed. This eplet is considered not antibody-verified because it is found on the bottom of the peptide-binding groove and therefore cannot induce antibody-formation.
			Navarette et al. 1997 HLA: Genetic diversity of HLA: Functional and Medical Implications Volume 1 - Proceedings of the Twelfth International Histocompatibility Workshop	X	This publication includes murine mAb analysis and is not peer-reviewed.This eplet is considered not antibody-verified because it is found on the bottom of the peptide-binding groove and therefore cannot induce antibody-formation.
DRB	13FE	13F 14E	Cai et al. 2006 Clin Transpl	B	This publication defines this eplet as 13F. This publication is not peer-reviewed.
			Cayrol et al. 1992 Tissue Antigens	D	
			Cayrol et al. 1995 Hum Immunol	D	
DRB	16Y	16Y 25R	HLA Epitope Registry website	B	This data is solely published on the HLA Epitope Registry website and is not peer-reviewed.
			Pistillo et al. 1991 Hum Immunol	C	
			Navarette et al. 1997 HLA: Genetic diversity of HLA: Functional and Medical Implications Volume 1 - Proceedings of the Twelfth International Histocompatibility Workshop	A1	This publication defines this eplet as 13G 14E 15C 16Y. This publication is not peer-reviewed.
			Nakayama et al. 1987 Hum Immunol	D	
			Cai et al. 2006 Clin Transpl	B	This publication defines the potential eplets as 13G/16Y. This publication is not peer-reviewed.
DRB	25Q	25Q 30L 14K	Nelson et al. 1989 Immunobiology of HLA Volume I: Histocompatibility Testing	D	This publication is not peer-reviewed.
			Kennedy et al. 1989 Immunobiology of HLA Volume I: Histocompatibility Testing	D	This publication is not peer-reviewed.
			Fu et al. 1995 Hum Immunol	D	
			Navarette et al. 1997 HLA: Genetic diversity of HLA: Functional and Medical Implications Volume 1 -	D	This publication is not peer-reviewed.

			Proceedings of the Twelfth International Histocompatibility Workshop		
			Cai et al. 2006 Clin Transpl	B	This publication defines the potential eplets as 11G/14K/25Q/30L. This publication is not peer-reviewed.
			Kramer et al. 2020 Am J Transplant	A1	This reference is not yet included in the HLA Epitope Registry for this eplet. This study defines this eplet as 14K 25Q.
DRB	25R	25R	Marsh et al. 1989 Immunol Today	D	
			Nelson et al. 1989 Immunobiology of HLA Volume I: Histocompatibility Testing	D	This publication is not peer-reviewed.
			Kennedy et al. 1989 Immunobiology of HLA Volume I: Histocompatibility Testing	D	This publication is not peer-reviewed.
			Inoko et al. 1992 HLA 1991 - Proceedings of the Eleventh International Histocompatibility Workshop and Conference – Volume 1	D	This publication is not peer-reviewed.
			Fu et al. 1995 Hum Immunol	D	
DRB	30C	30C	Wölpl et al. 1998 Tissue Antigens	X	This literature reference does not confirm the antibody verification of eplet 30C, because the human mAb UL-5A1 is peptide-dependent.
			Löffler et al. 1998 Eur J Immunogenet	X	This literature reference does not confirm the antibody verification of eplet 30C, because the human mAbs UL-5A1 and Fab-5A1 are peptide-dependent.
DRB	30RV	30R 31V 10E	Navarette et al. 1997 HLA: Genetic diversity of HLA: Functional and Medical Implications Volume 1 - Proceedings of the Twelfth International Histocompatibility Workshop	D	This publication is not peer-reviewed.
DRB	**31FY**	**31F 32Y**	Kramer et al. 2020 Am J Transplant	A1	This study defines this eplet as 31F 32Y 37Y/S. Eplet 31FY was not considered antibody-verified by the HLA Epitope Registry yet.
DRB	37L	37L	HLA Epitope Registry website	B	This data is solely published on the HLA Epitope Registry website and is not peer-reviewed.
			Cai et al. 2006 Clin Transpl	D	This reference is not yet included in the HLA Epitope Registry for this eplet. This publication is not peer-reviewed.
DRB	37YV	37Y 38V	Ge et al. 1995 Hum Immunol	X	This literature reference does not confirm antibody verification of eplet 37YV, but of eplet 31FY (A1 evidence).
			Marsh et al. 1992 HLA 1991 - Proceedings of the Eleventh International Histocompatibility Workshop and Conference – Volume 1	C	This publication is not peer-reviewed.
			Duquesnoy et al. 2016 Hum Immunol	X	This literature reference does not confirm antibody verification of eplet 37YV, but of eplet 31FY (B evidence).
			Bunce et al. 1990 Tissue Antigens	C	
DRB	47F	47F 48R	Cai et al. 2006 Clin Transpl	B	This publication defines this eplet as 47F. This publication is not peer-reviewed.
			Duquesnoy et al. 2016 Hum Immunol	B	
DRB	48Q	48Q 18L 25W 26N 41N 44L 81Y	Kosinski et al. 1986 Tissue Antigens	C	
			Knowles et al. 1986 J Immunol	D	
			Nelson et al. 1989 Immunobiology of HLA Volume I: Histocompatibility Testing	D	This publication is not peer-reviewed.
			Kennedy et al. 1989 Immunobiology of HLA Volume I: Histocompatibility Testing	D	This publication is not peer-reviewed.

DRB	51R	51R	Berte et al. 1989 Immunobiology of HLA Volume II: Immunogenetics and Histocompatibility	D	This publication is not peer-reviewed.
			Fuggle et al. 1989 Tissue Antigens	D	
			HLA Epitope Registry website	B	This data is solely published on the HLA Epitope Registry website and is not peer-reviewed.
DRB	57DE	57D 58E	HLA Epitope Registry website*	A1	SAB analysis of human mAb VR1H5 verifies eplet 57DE (Figure 4A).
			Hancock et al. 1988 Hum Immunol	C	
			Pistillo et al. 1991 Hum Immunol	C	
			Pistillo et al. 1992 Hum Immunol	C	
			Knowles 1989 Immunobiology of HLA Volume I: Histocompatibility Testing	D	This publication is not peer-reviewed.
			Marsh et al. 1989 Immunol Today	D	
			Nelson et al. 1989 Immunobiology of HLA Volume I: Histocompatibility Testing	D	This publication is not peer-reviewed.
			Klohe et al. 1992 Hum Immunol	X	The panel of cells used in this study is not sufficient for eplet analysis.
			Navarette et al. 1997 HLA: Genetic diversity of HLA: Functional and Medical Implications Volume 1 - Proceedings of the Twelfth International Histocompatibility Workshop	C	This publication is not peer-reviewed.
			Cai et al. 2006 Clin Transpl	D	This publication defines this eplet as 58E. This publication is not peer-reviewed.
DRB	57S	57S 58A 60Y	Cai et al. 2006 Clin Transpl	B	This publication defines this eplet as 57S. This reference is not yet included in the HLA Epitope Registry for this eplet and is not peer-reviewed.
DRB	57V	57V 58A 60S	HLA Epitope Registry website	B	This data is solely published on the HLA Epitope Registry website and is not peer-reviewed.
			Cai et al. 2006 Clin Transpl	B	This publication defines the potential eplets as 57V/60S. This reference is not yet included in the HLA Epitope Registry for this eplet and is not peer-reviewed.
DRB	70D	70D	Sadler et al. 1993 Tissue Antigens	D	
			Duquesnoy et al. 2016 Hum Immunol	B	
			Cai et al. 2006 Clin Transpl	B	This publication is not peer-reviewed.
DRB	70DA	70D 73A	Duquesnoy et al. 2016 Hum Immunol	B	
DRB	**70QA**	**70Q 73A**	Kramer et al. 2020 Am J Transplant	A1	Eplet 70QA is not considered antibody-verified yet by the HLA Epitope Registry.
DRB	70QT	70Q 77T	Drover et al. 1994 Hum Immunol	D	
			Inoko et al. 1992 HLA 1991 - Proceedings of the Eleventh International Histocompatibility Workshop and Conference – Volume 1	D	This publication is not peer-reviewed.
			Drover et al. 1992 HLA 1991 - Proceedings of the Eleventh International Histocompatibility Workshop and Conference – Volume 2	D	This publication is not peer-reviewed.
			Drover et al. 1994 Scand J Immunol	D	
DRB	70R	70R 71R	Cai et al. 2006 Clin Transpl	D	This publication defines this eplet as 70R. This publication is not peer-reviewed.
			HLA Epitope Registry website	B	This data is solely published on the HLA Epitope Registry website and is not peer-reviewed.
DRB	73A	73A 77T	Cayrol et al. 1992 Tissue Antigens	D	
			Fu et al. 1992 Hum Immunol	D	

DRB	74R	70Q 73G 74R	HLA Epitope Registry website*	A1	SAB analysis of human mAb BVK3D6 verifies eplet 74R (Figure S3C-D)
			Berte et al. 1989 Immunobiology of HLA Volume II: Immunogenetics and Histocompatibility	D	This publication is not peer-reviewed.
			Marsh et al. 1989 Immunol Today	D	
			Cai et al. 2006 Clin Transpl	B	This publication defines this eplet as 74R. This reference is not yet included in the HLA Epitope Registry for this eplet and is not peer-reviewed.
DRB	77N	73G 77N 78Y	Berte et al. 1989 Immunobiology of HLA Volume II: Immunogenetics and Histocompatibility	D	This publication is not peer-reviewed.
			Ballas et al. 1990 Tissue Antigens	D	
			Maurer et al. 1991 J Immunol	D	
			Klohe et al. 1993 Hum Immunol	D	
			Heyes et al. 1992 HLA 1991 - Proceedings of the Eleventh International Histocompatibility Workshop and Conference – Volume 2	D	This publication is not peer-reviewed.
			Cai et al. 2006 Clin Transpl	B	This publication defines this eplet as 77N. This publication is not peer-reviewed.
DRB	77T	77T	Madrigal et al. 1989 J Immunol	D	
			Maurer et al. 1992 HLA 1991 - Proceedings of the Eleventh International Histocompatibility Workshop and Conference – Volume 2	D	This publication is not peer-reviewed.
			Klohe et al. 1993 Hum Immunol	D	
			Cai et al. 2006 Am J Transplant	A2	
DRB	96EV	96E 98K 180V	Marrari et al. 2009 Hum Immunol	B	
			Loh et al. 1993 Tissue Antigens	D	
			Cai et al. 2006 Am J Transplant	B	This publication defines this eplet as 96E.
DRB	96HK	96H 98K 120S	HLA Epitope Registry website*	X	SAB analysis of human mAb RTLK1E2 does not confirm eplet 96HK, but verifies eplet 149H (Figure 4C).
			HLA Epitope Registry website	B	This data is solely published on the HLA Epitope Registry website and is not peer-reviewed.
			Navarette et al. 1997 HLA: Genetic diversity of HLA: Functional and Medical Implications Volume 1 - Proceedings of the Twelfth International Histocompatibility Workshop	D	This publication is not peer-reviewed.
			Cai et al. 2006 Clin Transpl	X	This publication does not provide evidence for the antibody verification of eplet 96HK.
DRB	96Y	96Y	Maurer et al. 1991 J Immunol	D	
			Drover et al. 1994 Hum Immunol	D	
			Inoko et al. 1992 HLA 1991 - Proceedings of the Eleventh International Histocompatibility Workshop and Conference – Volume 1	D	This publication is not peer-reviewed.
			Cai et al. 2006 Clin Transpl	B	This publication defines the potential eplets as 13H/33H/96Y/180L. This publication is not peer-reviewed.
DRB	98E	98E	HLA Epitope Registry website	B	This data is solely published on the HLA Epitope Registry website and is not peer-reviewed.
			Cai et al. 2006 Clin Transpl	B	This publication is not peer-reviewed.
DRB	98ES	98E 120S	HLA Epitope Registry website	B	This data is solely published on the HLA Epitope Registry website and is not peer-reviewed.
			Duquesnoy et al. 2016 Hum Immunol	B	

			Cai et al. 2006 Clin Transpl	B	This publication defines this eplet as 78V. This publication is not peer-reviewed.
			Kramer et al. 2020 Am J Transplant	A1	This reference is not yet included in the HLA Epitope Registry for this eplet. This study defined this eplet as 78V *or* 96H 98E 120S.
DRB	98Q	96H 98Q 120S	Taylor et al. 1989 Immunobiology of HLA Volume I: Histocompatibility Testing	C	This publication is not peer-reviewed.
			Kolstad et al. 1988 Tissue Antigens	C	
			Cai et al. 2006 Clin Transpl	D	This publication defines this eplet as 98Q. This publication is not peer-reviewed.
DRB	104A	104A	Cai et al. 2006 Clin Transpl	D	This publication is not peer-reviewed.
			Cai et al. 2006 Am J Transplant	B	
DRB	108T	108T	Van den Berg-Loonen et al. 1997 HLA: Genetic diversity of HLA: Functional and Medical Implications Volume 1 - Proceedings of the Twelfth International Histocompatibility Workshop	C	This publication is not peer-reviewed.
			Cai et al. 2006 Clin Transpl	D	This publication defines the potential eplets as 9Q/108T. This publication is not peer-reviewed.
DRB	142M	142M	Bodmer et al. 1977 Histocompatibility testing 1977	C	This publication is not peer-reviewed.
			Nelson et al. 1989 Immunobiology of HLA Volume I: Histocompatibility Testing	D	This publication is not peer-reviewed.
			Navarette et al. 1997 HLA: Genetic diversity of HLA: Functional and Medical Implications Volume 1 - Proceedings of the Twelfth International Histocompatibility Workshop	D	This publication is not peer-reviewed.
			Cai et al. 2006 Clin Transpl	B	This publication defines the potential eplets as 11P/13R/133L/142M. This reference is not yet included in the HLA Epitope Registry for this eplet
DRB	**149H**	**149H**	*	A1	SAB analysis of human mAb RTLK1E2 verifies eplet 149H (Figure 4C). Eplet 149H is not considered antibody-verified yet by the HLA Epitope Registry.
			Cai et al. 2006 Clin Transpl	B	This reference is not yet included in the HLA Epitope Registry for this eplet. This publication is not peer-reviewed.
DRB	181M	181M	Fu et al. 1992 Hum Immunol	D	
			Fu et al. 1995 Hum Immunol	D	
			Cai et al. 2006 Am J Transplant	B	
			Cai et al. 2006 Clin Transpl	B	This reference is not yet included in the HLA Epitope Registry for this eplet. This publication is not peer-reviewed.
DQB	45EV	45E 46V 47Y	Schreuder et al. 1986 Hum Immunol	D	
			Kenter et al. 1989 Hum Immunol	D	
			Maeda et al. 1986 Tissue Antigens	D	
			Navarette et al. 1997 HLA: Genetic diversity of HLA: Functional and Medical Implications Volume 1 - Proceedings of the Twelfth International Histocompatibility Workshop	D	This publication is not peer-reviewed.
			Deng et al. 2006 Clin Transpl	B	This publication defines this eplet as 45E. This publication is not peer-reviewed.
			El-Awar et al. 2013 Hum Immunol	A2	This publication defines this eplet as 45E.
			Deng et al. 2008 Transplantation	B	This publication defines this eplet as 45E. This reference is not yet included in the HLA Epitope Registry for this eplet.
			Bezstarosti et al. 2022 Front Immunol	A1	This reference is not yet included in the HLA Epitope Registry for this eplet.
DQB	45GV	45G 46V	Schreuder et al. 1986 Hum Immunol	D	
			Radka et al. 1989 Hum Immunol	D	

			Kenter et al. 1989 Hum Immunol	D	
			Nordwig et al. 1991 Hum Immunol	D	
			Viken et al. 1995 Tissue Antigens	D	
			Petersen et al. 1992 J Immunol Methods	D	
			El-Awar et al. 2013 Hum Immunol	A2	
			Deng et al. 2008 Transplantation	B	This publication defines this eplet as 45G+46V. This reference is not yet included in the HLA Epitope Registry for this eplet.
DQB	46VY	46V 52P 28T	Marsh et al. 1989 Immunol Today	D	
			El-Awar et al. 2013 Hum Immunol	A2	This publication defines this eplet as 52P.
			Deng et al. 2006 Clin Transpl	B	This publication defines the potential eplets as 28T/46V/52P. This publication is not peer-reviewed.
			Deng et al. 2008 Transplantation	A2	This publication defines the potential eplets as 28T/46V/52P.
DQB	52LL	52L 55L 28S 30S 37I	Pistillo et al. 1986 Hum Immunol	C	
			Pistillo et al. 1992 Hum Immunol	X	The panel of cells used in this study is not sufficient for eplet analysis.
			Viken et al. 1995b Hum Immunol	D	
			Viken et al. 1995a Hum Immunol	D	
			El-Awar et al. 2013 Hum Immunol	A2	This publication defines this eplet as 52L. This reference is not yet included in the HLA Epitope Registry for this eplet.
			Deng et al. 2008 Transplantation	B	This publication defines this eplet as 28S/30S/37I/52L/55L. This reference is not yet included in the HLA Epitope Registry for this eplet.
			Bezstarosti et al. 2022 Front Immunol	A1	This reference is not yet included in the HLA Epitope Registry for this eplet.
DQB	52PQ	53Q 89G 90I	Hansen et al. 1987 Hum Immunol	C	
			Koning et al. 1985 Tissue Antigens	D	
			Marsh et al. 1989 Immunol Today	D	
			Dessi et al. 1990 Hum Immunol	C	
			Navarette et al. 1997 HLA: Genetic diversity of HLA: Functional and Medical Implications Volume 1 - Proceedings of the Twelfth International Histocompatibility Workshop	D	This publication is not peer-reviewed.
			Nelson et al. 1989 Immunobiology of HLA Volume I: Histocompatibility Testing	D	This publication is not peer-reviewed.
			El-Awar et al. 2013 Hum Immunol	A2	This publication defines this eplet as 84E.
			Deng et al. 2008 Transplantation	B	This publication defines the potential eplets as 84E/85V/86A/89G/90I/221Q. This reference is not yet included in the HLA Epitope Registry for this eplet.
DQB	55PP	55P 56P	Marsh et al. 1989 Immunol Today	D	
			Nelson et al. 1989 Immunobiology of HLA Volume I: Histocompatibility Testing	D	This publication is not peer-reviewed.
			Navarette et al. 1997 HLA: Genetic diversity of HLA: Functional and Medical Implications Volume 1 - Proceedings of the Twelfth International Histocompatibility Workshop	D	This publication is not peer-reviewed.
			Viken et al. 1995c Hum Immunol	X	This reference does not provide evidence for the antibody verification of eplet 55PP.
			Deng et al. 2008 Transplantation	B	This publication defines this eplet as 55P.
			El-Awar et al. 2013 Hum Immunol	A2	This publication defines this eplet as 55P.
			Bezstarosti et al. 2022 Front Immunol	A1	This reference is not yet included in the HLA Epitope Registry for this eplet.
DQB	55R	55R	Kolstad et al. 1989b Hum Immunol	C	
			Marsh et al. 1989 Immunol Today	D	

			Nelson et al. 1989 Immunobiology of HLA Volume I: Histocompatibility Testing	C/D	This publication is not peer-reviewed.
			Kennedy et al. 1989 Immunobiology of HLA Volume I: Histocompatibility Testing	C/D	This publication is not peer-reviewed.
			Vitale et al. 1992 Hum Immunol	D	
			Sánchez et al. 1993 Hum Immunol	C	
			Navarette et al. 1997 HLA: Genetic diversity of HLA: Functional and Medical Implications Volume 1 - Proceedings of the Twelfth International Histocompatibility Workshop	D	This publication is not peer-reviewed.
			El-Awar et al. 2013 Hum Immunol	A2	
			HLA Epitope Registry website	B	This data is solely published on the HLA Epitope Registry website and is not peer-reviewed.
			Deng et al. 2008 Transplantation	B	This publication defines this eplet as 52P+55R. This reference is not yet included in the HLA Epitope Registry for this eplet.
			Bezstarosti et al. 2022 Front Immunol	A1	This reference is not yet included in the HLA Epitope Registry for this eplet.
DQB	56L	56L 71D	Ishikawa et al. 1987 Immunogenetics	D	
			Matsuki et al. 1987 Hum Immunol	B	
			Marsh et al. 1989 Immunol Today	D	
			Kennedy et al. 1989 Immunobiology of HLA Volume I: Histocompatibility Testing	D	This publication is not peer-reviewed.
			Navarette et al. 1997 HLA: Genetic diversity of HLA: Functional and Medical Implications Volume 1 - Proceedings of the Twelfth International Histocompatibility Workshop	D	This publication is not peer-reviewed.
			Deng et al. 2008 Transplantation	B	This publication defines this eplet as 56L. This reference is not yet included in the HLA Epitope Registry for this eplet.
DQB	57V	57V	El-Awar et al. 2013 Hum Immunol	X	This literature reference does not provide evidence for the antibody verification of eplet 57V.
			HLA Epitope Registry website	B	This data is solely published on the HLA Epitope Registry website and is not peer-reviewed.
DQB	74S	74S 26G	Ge et al. 1994 Hum Immunol	A1	
			Duquesnoy et al. 2008 Transpl Immunol	B	
DQB	77R	75V 77R	El-Awar et al. 2013 Hum Immunol	A2	This publication defines this eplet as 77R.
DQB	77T	77T	El-Awar et al. 2013 Hum Immunol	A2	
DQB	84QL	84Q 86E 87L 89T 90T 125A	Vitale et al. 1992 Hum Immunol	D	
			El-Awar et al. 2013 Hum Immunol	A2	This publication defines this eplet as 84Q.
			Radka et al. 1985 Hum Immunol	D	
			Deng et al. 2008 Transplantation	A2	This publication defines the potential eplets as 84Q/85L/86E/87L/89T/220H/221H.
			Bezstarosti et al. 2022 Front Immunol	A1	This reference is not yet included in the HLA Epitope Registry for this eplet.
DQB	87F	87F	HLA Epitope Registry website	B	This data is solely published on the HLA Epitope Registry website and is not peer-reviewed.
DQB	87Y	87Y	HLA Epitope Registry website	B	This data is solely published on the HLA Epitope Registry website and is not peer-reviewed.
DQB	116I	116I 125S	Marsh et al. 1989 Immunol Today	D	
			Deng et al. 2006 Clin Transpl	B	This publication defines the potential eplets as 70G+71A/116I/125S. This publication is not peer-reviewed.

			El-Awar et al. 2013 Hum Immunol	A2	This publication defines this eplet as 125S.
			Deng et al. 2008 Transplantation	B	This publication defines the potential eplets as 70G+71A/116I/125S. This reference is not yet included in the HLA Epitope Registry for this eplet.
DQB	125SQ	125S 126Q	El-Awar et al. 2013 Hum Immunol	A2	
			El-Awar et al. 2017 J Immunol Res	X	This literature reference does not provide any evidence for antibody verification of eplet 125SQ.
DQB	182N	182N	Navarette et al. 1997 HLA: Genetic diversity of HLA: Functional and Medical Implications Volume 1 - Proceedings of the Twelfth International Histocompatibility Workshop	D	This publication is not peer-reviewed.
			El-Awar et al. 2013 Hum Immunol	A2	
			Deng et al. 2008 Transplantation	B	This publication defines the potential eplets as 77T+84Q/ 77T+85L/ 77T+ 86E/77T+ 87L/ 182N.
			Bezstarosti et al. 2022 Front Immunol	A1	This reference is not yet included in the HLA Epitope Registry for this eplet.
DQB	182S	182S	El-Awar et al. 2013 Hum Immunol	A2	This publication defines this eplet as 140A.
DQA	2D	2D 199A	Youngs et al. 2018 Transpl Immunol	B	
DQA	40E	40E	HLA Epitope Registry website	B	This data is solely published on the HLA Epitope Registry website and is not peer-reviewed.
DQA	40GR	40G 47C 50V 51L	El-Awar et al. 2013 Hum Immunol	A2	This publication defines this eplet as 40G.
			Deng et al. 2008 Transplantation	A2	This publication defines the potential eplets as 40G/47C.
			Tambur et al. 2010 Transplantation	B	
			Duquesnoy et al. 2008 Transpl Immunol	B	
DQA	47KHL	47K 52H 54L	El-Awar et al. 2013 Hum Immunol	A2	This publication defines this eplet as 52H.
			Deng et al. 2008 Transplantation	B	This publication defines the potential eplets as 47K/52H/54L.
			Duquesnoy et al. 2008 Transpl Immunol	B	
DQA	52SK	52S 53K 11C 18F 45A 64R 66M 69A 80Y	Chersi et al. 1989 Z Naturforsch C J Biosci	D	
			HLA Epitope Registry website	B	This data is solely published on the HLA Epitope Registry website and is not peer-reviewed.
DQA	61FT	61F 64T 55R	HLA Epitope Registry website	B	This data is solely published on the HLA Epitope Registry website and is not peer-reviewed.
DQA	75S	75S 156L 163E 175K	Kolstad et al. 1989b Hum Immunol	C	
			Tambur et al. 2010 Transplantation	B	
DQA	76V	76V 26S 47Q 187T	HLA Epitope Registry website	B	This data is solely published on the HLA Epitope Registry website and is not peer-reviewed.
			Deng et al. 2008 Transplantation	B	This publication defines the potential eplet as 26S/47Q/ 56R/187T. This reference is not yet included in the HLA Epitope Registry for this eplet
DPB	35FV	35F 36V	Marshall et al. 1992 HLA 1991 - Proceedings of the Eleventh International Histocompatibility Workshop and Conference – Volume 2	D	This publication is not peer-reviewed.
			Marshall et al. 1998 Hum Immunol	D	
			Youngs 2004 ASHI Quarterly	B	This publication is not peer-reviewed.
			Billen et al. 2010 Tissue Antigens	B	
DPB	56A	56A	HLA Epitope Registry website	B	This data is solely published on the HLA Epitope Registry website and is not peer-reviewed.
			Cano et al. 2009 Hum Immunol	B	
DPB	56E	55D 56E	HLA Epitope Registry website*	X	SAB analysis of human mAb VR1H5 verifies HLA-DR eplet 58E and is cross-reactive with HLA-DP eplet 56E. mAb VR1H5 does not provide evidence for HLA-DP eplet 56E as inducer of the antibody response (Figure 4A).

			*	A1	SAB analysis of human mAb RTLK10E12 verifies eplet 56E (Figure 4B).
			Mazzoleni et al. 1989 Immunogenetics	C	
			Pistillo et al. 1992 HLA 1991 - Proceedings of the Eleventh International Histocompatibility Workshop and Conference – Volume 2	A1	This publication is not peer-reviewed.
			Marshall et al. 1998 Hum Immunol	D	
			Cano et al. 2009 Hum Immunol	B	This reference is not yet included in the HLA Epitope Registry for this eplet.
DPB	56EE	55D 56E 57E	Deng et al. 2007 Clin Transpl	B	This publication is not peer-reviewed.
DPB	57D	55D 56E 57D	Marshall et al. 1998 Hum Immunol	D	
			Tongio et al. 1997 HLA: Genetic diversity of HLA: Functional and Medical Implications Volume 2 - Proceedings of the Twelfth International Histocompatibility Conference	D	This publication is not peer-reviewed.
			Deng et al. 2007 Clin Transpl	B	This publication is not peer-reviewed.
			Billen et al. 2010 Tissue Antigens	B	
			HLA Epitope Registry website	B	This data is solely published on the HLA Epitope Registry website and is not peer-reviewed.
DPB	69E	69E	Arroyo et al. 1995 Hum Immunol	D	
DPB	84DEAV	84D 85E 86A 87V	HLA Epitope Registry website*	A1	SAB analysis of human mAb TL3B6 verifies eplet 84DEAV (Figure S3A-B).
			Deng et al. 2007 Clin Transpl	A2	This publication is not peer-reviewed.
			Kolstad et al. 1989a Hum Immunol	C	
			Marshall et al. 1998 Hum Immunol	D	
			Tongio et al. 1997 HLA: Genetic diversity of HLA: Functional and Medical Implications Volume 2 - Proceedings of the Twelfth International Histocompatibility Conference	D	This publication is not peer-reviewed.
			Youngs 2004 ASHI Quarterly	B	This publication is not peer-reviewed.
			Duquesnoy et al. 2008 Transpl Immunol	B	
			Billen et al. 2010 Tissue Antigens	B	
			Cano et al. 2009 Hum Immunol	B	
DPB	85GPM	85G 86P 87M	Viken et al. 1989 Tissue Antigens	D	
			Marshall et al. 1998 Hum Immunol	D	
			Inoko et al. 1992 HLA 1991 - Proceedings of the Eleventh International Histocompatibility Workshop and Conference – Volume 1	D	This publication is not peer-reviewed.
			Tongio et al. 1997 HLA: Genetic diversity of HLA: Functional and Medical Implications Volume 2 - Proceedings of the Twelfth International Histocompatibility Conference	D	This publication is not peer-reviewed.
			Youngs 2004 ASHI Quarterly	B	This publication is not peer-reviewed.
			Billen et al. 2010 Tissue Antigens	B	
			Cano et al. 2009 Hum Immunol	B	
			HLA Epitope Registry website	B	This data is solely published on the HLA Epitope Registry website and is not peer-reviewed.
			Kramer et al. Manuscript in preparation	A1	

			Deng et al. 2007 Clin Transpl	B	This publication defines this eplet as 85G+86G+86P +87M. This reference is not yet included in the HLA Epitope Registry for this eplet and is not peer-reviewed.
DPB	96K	96K 170I	HLA Epitope Registry website	B	This data is solely published on the HLA Epitope Registry website and is not peer-reviewed.
DPA	50Q	50Q	Duquesnoy et al. 2008 Transpl Immunol	B	
			HLA Epitope Registry website	B	This data is solely published on the HLA Epitope Registry website and is not peer-reviewed.
DPA	50R	50R	Marshall et al. 1998 Hum Immunol	D	
			Heyes et al. 1986 Proc Natl Acad Sci U S A	D	
			Young et al. 1988 Hum Immunol	D	
			HLA Epitope Registry website	B	This data is solely published on the HLA Epitope Registry website and is not peer-reviewed.
			Thammanichanond et al. 2018 Transplant Proc	B	

[1]A1, Human monoclonal antibody + single antigen bead (SAB) assay and/or complement dependent cytotoxicity assay (CDC) with high resolution HLA typed cells; A2, Adsorption and elution studies + SAB and/or CDC with high resolution HLA typed cells; B, Patient serum tested in SAB; C, Human monoclonal antibody or adsorption and elution studies or patient sera tested with serologically typed cells; D, Other species (e.g. murine monoclonal antibody).
*Single antigen bead analysis of this human mAb is provided in this paper.
Bold: Eplets that currently not antibody-verified in the HLA Epitope Registry.
<u>Underlined:</u> The residues of these eplets exceed the 3.5 Ångstrom radius and we therefore propose to consider these eplets as antibody-verified reactivity patterns. See Table 7 for proposed definitions and rationale.

Supplementary Table 4. Residue differences in reactivity patterns on Common HLA alleles

Reactivity pattern	Allele*	Residues	Common in population
44K / 150V / 158V	A*01:06	44K / 150V / **158A**	MENA
	A*24:22	**44R** / **150A** / 158V	HIS, NAM
74S / 26G	DQB1*03:05	**74E** / 26G	AFA, API, EURO, MENA, HIS, NAM, UNK
	DQB1*03:25	**74E** / 26G	NAM
53L / 84Q / 85L / 86E / 87L / 89T / 90T / 125A / 220H / 221H	DQB1*03:09	53L / 84Q / 85L / 86E / 87L / 89T / 90T / 125A / 220H / **221R**	AFA, NAM
52P + 53L / 140T / 182N	DQB1*03:09	52P + 53L / 140T / **182P**	AFA, NAM

*Common alleles in the CIWD database (version 3.0.0) (Hurley et al. HLA. 2020 Jun;95(6):516-531. doi: 10.1111/tan.13811.)

AFA; African/African American, API; Asian/Pacific Islands, EURO; European/European descent, MENA; Middle East/North coast of Africa, HIS; South or Central America/Hispanic/Latino, NAM; Native American populations, UNK; Unknown/Not asked/Multiple ancestries/Other.

References

HLA Epitope Registry website.

Adeyi, OA, AL Girnita, J Howe, M Marrari, Y Awadalla, M Askar, et al. Serum analysis after transplant nephrectomy reveals restricted antibody specificity patterns against structurally defined HLA class I mismatches. *Transpl Immunol* (2005) 14(1): 53-62.

Akaza, T, N El-Awar, A Nguyen, J Kitawaki and P Terasaki. HLA class I epitopes: C-locus. *Clin Transpl* (2006): 95-102.

Arroyo, J, AM Alvarez, C Nombela and M Sánchez-Pérez. The role of HLA-DP beta residue 69 in the definition of antibody-binding epitopes. *Hum Immunol* (1995) 43(3): 219-226.

Bachelet, T, L Couzi, G Guidicelli, K Moreau, D Morel, P Merville, et al. Anti-Cw donor-specific alloantibodies can lead to positive flow cytometry crossmatch and irreversible acute antibody-mediated rejection. *Am J Transplant* (2011) 11(7): 1543-1544.

Ballas, M, TH Eiermann, A Wolpl and SF Goldmann. Mapping of an HLA-DRw52-associated determinant on DR beta 1 molecules. *Tissue Antigens* (1990) 36(5): 187-193.

Berte, CC, J Gorski, W Reith and B Mach. Epitope Mapping of HLA-DR Antigens with the Use of DNA-Transfected Cells. In: B Dupont, editor. *Immunobiology of HLA Volume II: Immunogenetics and Histocompatibility*. New York: Springer-Verlag (1989). p. 245-247.

Bezstarosti, S, CSM Kramer, MEI Franke-van Dijk, M Vergunst, KH Bakker, M Uyar-Mercankaya, et al. HLA-DQ-Specific Recombinant Human Monoclonal Antibodies Allow for In-Depth Analysis of HLA-DQ Epitopes. *Front Immunol* (2022) 12: 761893.

Billen, EV, MH Christiaans, Doxiadis, II, CE Voorter and EM van den Berg-Loonen. HLA-DP antibodies before and after renal transplantation. *Tissue Antigens* (2010) 75(3): 278-285.

Bodmer, JG, P Pickbourne and S Richards. Joint Report: I a Serology In: WF Bodmer, JR Batchelor, JG Bodmer, H Festenstein and PJ Morris, editors. *Histocompatibility testing 1977*. Copenhagen: Munksgaard (1977). p. 35-84.

Bunce, M, PM Sutton, A Ting and PJ Morris. The production of a human monoclonal antibody defining a split of HLA-DRw13 (DRw13b). *Tissue Antigens* (1990) 36(3): 100-102.

Cai, J, S Kohanof and PI Terasaki. HLA-DR antibody epitopes. *Clin Transpl* (2006): 103-114.

Cai, J, PI Terasaki, Q Mao, T Pham, N El-Awar, JH Lee, et al. Development of nondonor-specific HLA-DR antibodies in allograft recipients is associated with shared epitopes with mismatched donor DR antigens. *Am J Transplant* (2006) 6(12): 2947-2954.

Cano, P and M Fernández-Viña. Two sequence dimorphisms of DPB1 define the immunodominant serologic epitopes of HLA-DP. *Hum Immunol* (2009) 70(10): 836-843.

Cauquil, B, G Dautin and RJ Duquesnoy. Case report: A transplant candidate with unexpected serum reactivity against the 45KE eplet on HLA-B alleles. *Hla* (2018) 92(4): 231-232.

Cayrol, C, F Moro, E Sommer, J Tkaczuk, E Ohayon and A Cambon-Thomsen. New polymorphic HLA-DR epitopes recognized by three monoclonal antibodies produced against DR103 transfected L cells. *Tissue Antigens* (1992) 40(4): 197-203.

Cayrol, C, J Tkaczuk, E Sommer and A Cambon-Thomsen. A subset of HLA-DR9 molecules is detected by a polymorphic monoclonal antibody on lymphoblastoid cell lines but not on peripheral blood lymphocytes. *Hum Immunol* (1995) 44(1): 19-27.

Chersi, A, TF Romano and F Chillemi. Preparation and isolation of antibodies to human MHC class II alpha chains by aid of synthetic peptides. *Z Naturforsch C J Biosci* (1989) 44(9-10): 813-818.

De Vito, LD, BP Mason, E Jankowska-Gan, KT Hogan, JW Guo, CT Lutz, et al. Epitope fine specificity of human anti-HLA-A2 antibodies. Identification of four epitopes including a haptenlike epitope on HLA-A2 at lysine 127. *Hum Immunol* (1993) 37(3): 165-177.

Deng, CT, J Cai, M Ozawa and N El-Awar. HLA class II DP epitopes. *Clin Transpl* (2007): 195-202.

Deng, CT, J Cai, C Tarsitani, N El-Awar, N Lachmann and M Ozawa. HLA class II DQ epitopes. *Clin Transpl* (2006): 115-122.

Deng, CT, N El-Awar, M Ozawa, J Cai, N Lachmann and PI Terasaki. Human leukocyte antigen class II DQ alpha and beta epitopes identified from sera of kidney allograft recipients. *Transplantation* (2008) 86(3): 452-459.

Dessi, V, B Sanchez, M Garzon, R Magarino, MD Maldonado and A Nunez-Roldan. Characterization of three human monoclonal antibodies specific for polymorphic class I and class II HLA antigens. *Hum Immunol* (1990) 27(4): 323-332.

Drover, S, RW Karr, XT Fu and WH Marshall. Analysis of monoclonal antibodies specific for unique and shared determinants on HLA-DR4 molecules. *Hum Immunol* (1994) 40(1): 51-60.

Drover, S, WH Marshall and R Karr. Contribution of residues 70 and 73 of HLA-DRB1 chains to the binding of monoclonal antibody NFLD.D10. In: K Tsuji, M Aizawa and T Sasazuki, editors. *HLA 1991 - Proceedings of the Eleventh International Histocompatibility Workshop and Conference – Volume 2*. Oxford: Oxford University Press (1992). p. 408-410.

Drover, S, WH Marshall, WW Kwok, GT Nepom and RW Karr. Amino acids in the peptide-binding groove influence an antibody-defined, disease-associated HLA-DR epitope. *Scand J Immunol* (1994) 39(6): 539-550.

Duquesnoy, RJ, Y Awadalla, J Lomago, L Jelinek, J Howe, D Zern, et al. Retransplant candidates have donor-specific antibodies that react with structurally defined HLA-DR,DQ,DP epitopes. *Transpl Immunol* (2008) 18(4): 352-360.

Duquesnoy, RJ, G Honger, I Hosli, M Marrari and S Schaub. Detection of newly antibody-defined epitopes on HLA class I alleles reacting with antibodies induced during pregnancy. *Int J Immunogenet* (2016) 43(4): 200-208.

Duquesnoy, RJ, G Honger, I Hosli, M Marrari and S Schaub. Identification of epitopes on HLA-DRB alleles reacting with antibodies in sera from women sensitized during pregnancy. *Hum Immunol* (2016) 77(2): 214-222.

Duquesnoy, RJ and M Marrari. Detection of antibodies against HLA-C epitopes in patients with rejected kidney transplants. *Transpl Immunol* (2011) 24(3): 164-171.

Duquesnoy, RJ, M Marrari, L Jelenik, A Zeevi, FH Claas and A Mulder. Structural aspects of HLA class I epitopes reacting with human monoclonal antibodies in Ig-binding, C1q-binding and lymphocytotoxicity assays. *Hum Immunol* (2013) 74(10): 1271-1279.

Duquesnoy, RJ, M Marrari, A Mulder, FH Claas, J Mostecki and I Balazs. Structural aspects of human leukocyte antigen class I epitopes detected by human monoclonal antibodies. *Hum Immunol* (2012) 73(3): 267-277.

El-Awar, N, V Jucaud and A Nguyen. HLA Epitopes: The Targets of Monoclonal and Alloantibodies Defined. *J Immunol Res* (2017) 2017: 3406230.

El-Awar, N, JH Lee, C Tarsitani and PI Terasaki. HLA class I epitopes: recognition of binding sites by mAbs or eluted alloantibody confirmed with single recombinant antigens. *Hum Immunol* (2007) 68(3): 170-180.

El-Awar, N, A Nguyen, K Almeshari, M Alawami, F Alzayer, M Alharbi, et al. HLA class II DQA and DQB epitopes: recognition of the likely binding sites of HLA-DQ alloantibodies eluted from recombinant HLA-DQ single antigen cell lines. *Hum Immunol* (2013) 74(9): 1141-1152.

El-Awar, N, PI Terasaki, J Cai, CT Deng, M Ozawa, A Nguyen, et al. Epitopes of HLA-A, B, C, DR, DQ, DP and MICA antigens. *Clin Transpl* (2009): 295-321.

El-Awar, N, PI Terasaki, A Nguyen, M Lias and N Conger. New HLA class I epitopes defined by murine monoclonal antibodies. *Hum Immunol* (2010) 71(5): 456-461.

El-Awar, NR, T Akaza, PI Terasaki and A Nguyen. Human leukocyte antigen class I epitopes: update to 103 total epitopes, including the C locus. *Transplantation* (2007) 84(4): 532-540.

Fernandez-Vina, M, M Maiers, M Gutierrez, A Mulder, JH Lee, J Stoddard, et al. 13th IHWS Serology and HLA Phenotypes Joint Report. In: JA Hansen, editor. *Immunobiology of the Human MHC - Proceedings of the 13th International Histocompatibility Workshop and Conference - Volume I*. Seatttle IHWG Press (2006). p. 890-931.

Fu, XT, S Drover, WH Marshall and RW Karr. HLA-DR residues accessible under the peptide-binding groove contribute to polymorphic antibody epitopes. *Hum Immunol* (1995) 43(4): 243-250.

Fu, XT and RW Karr. HLA-DR alpha chain residues located on the outer loops are involved in nonpolymorphic and polymorphic antibody-binding epitopes. *Hum Immunol* (1994) 39(4): 253-260.

Fu, XT, WY Yu, C Alber, C Benson, R Watts, H Nordwig, et al. Identification of residues involved in polymorphic antibody binding epitopes on HLA-DR molecules. *Hum Immunol* (1992) 33(1): 47-56.

Fuggle, SV, C Carter and PJ Morris. Monoclonal antibody definition of the DRB3 allele, HLA-Dw25. *Tissue Antigens* (1989) 34(3): 149-157.

Ge, J, A Bratlie and K Hannestad. A human hybridoma monoclonal antibody (TrJ11) recognizing a new HLA-DR epitope shared by DR4, DR8, DR11, and DRB1*1303. *Hum Immunol* (1995) 42(1): 27-34.

Ge, J and K Hannestad. A cytotoxic human hybridoma monoclonal antibody (TrJ5) specific for HLA-B38(16) and -B39(16). *Hum Immunol* (1993) 36(3): 168-171.

Ge, J and K Hannestad. A cytotoxic human hybridoma monoclonal antibody (TrJ6) defining an epitope expressed by HLA-DQ4 and -DQ5. *Hum Immunol* (1994) 39(2): 106-112.

Hancock, RJ, A Martin, GJ Laundy, J Smythe, I Roberts, H Cooke, et al. Production of monoclonal human antibody to HLA-DR5 (DRw11) by mouse/human heterohybridomas. *Hum Immunol* (1988) 22(2): 135-142.

Hansen, T, A Kolstad, G Mathisen and K Hannestad. A human-human hybridoma (Tr7E2) producing cytotoxic antibody to HLA-DQw1. *Hum Immunol* (1987) 20(4): 307-320.

Heyes, J, P Austin, J Bodmer, W Bodmer, A Madrigal, MC Mazzilli, et al. Monoclonal antibodies to HLA-DP-transfected mouse L cells. *Proc Natl Acad Sci U S A* (1986) 83(10): 3417-3421.

Heyes, JM, H Inoko, J Trowsdale, A Sadler, SG Marsh and J Bodmer. Fine mapping of Eleventh International Histocompatibility Workshop HLA class II monoclonal antibodies using transfectants. In: K Tsuji, M Aizawa and T Sasazuki, editors. *HLA 1991 - Proceedings of the Eleventh International Histocompatibility Workshop and Conference – Volume 2*. Oxford: Oxford University Press (1992). p. 400-404.

Hogan, KT, C Clayberger, EJ Bernhard, SF Walk, JP Ridge, P Parham, et al. Identification by site-directed mutagenesis of amino acid residues contributing to serologic and CTL-defined epitope differences between HLA-A2.1 and HLA-A2.3. *J Immunol* (1988) 141(7): 2519-2525.

Inoko, H, JG Bodmer, JM Heyes, S Drover, J Trowsdale and WH Marshall. Joint report on the transfectant/monoclonal antibody component. In: K Tsuji, M Aizawa and T Sasazuki, editors. *HLA 1991 - Proceedings of the Eleventh International Histocompatibility Workshop and Conference – Volume 1*. Oxford: Oxford University Press (1992). p. 919-930.

Ishikawa, N, H Kojima, T Nakayama, H Kunikane, S Hawkin, Y Fukasawa, et al. Detection of a novel HLA-DQ specificity: serological and immunochemical analyses by a monoclonal antibody. *Immunogenetics* (1987) 26(3): 143-149.

Kennedy, LJ, SGE Marsh and J Bodmer. Cytotoxic Monoclonal Antibodies In: B Dupont, editor. *Immunobiology of HLA Volume I: Histocompatibility Testing*. New York: Springer-Verlag (1989). p. 301-305.

Kenter, MJ, JD Anholts, GM Schreuder, MC van Eggermond, GM Ghyselen, JJ van Rood, et al. Unambiguous typing for HLA-DQ TA10 and 2B3 specificities using specific oligonucleotide probes. *Hum Immunol* (1989) 24(1): 65-73.

Klohe, E, XT Fu, M Ballas and RW Karr. HLA-DR beta chain residues that are predicted to be located in the floor of the peptide-binding groove contribute to antibody-binding epitopes. *Hum Immunol* (1993) 37(1): 51-58.

Klohe, E, MP Pistillo, GB Ferrara, NE Goeken, NS Greazel and RW Karr. Critical role of HLA-DR beta 1 residue 58 in multiple polymorphic epitopes recognized by xenogeneic and allogeneic antibodies. *Hum Immunol* (1992) 35(1): 18-28.

Knowles, RW. Structural Polymorphism of the HLA Class II α and β Chains: Summary of the 10th Workshop 2-D Gel Analysis. In: B Dupont, editor. *Immunobiology of HLA Volume I: Histocompatibility Testing*. New York: Springer-Verlag (1989). p. 365-380.

Knowles, RW, N Flomenberg, K Horibe, R Winchester, SF Radka and B Dupont. Complexity of the supertypic HLA-DRw53 specificity: two distinct epitopes differentially expressed on one or all of the DR beta-chains depending on the HLA-DR allotype. *J Immunol* (1986) 137(8): 2618-2626.

Kolstad, A and K Hannestad. A supertypic HLA-DP specificity defined by two human-human hybridoma antibodies (TrB50; TrE11). *Hum Immunol* (1989a) 25(4): 247-256.

Kolstad, A, T Hansen and K Hannestad. A cytotoxic human-human hybridoma antibody (TrH6) specific for HLA-DRw52. *Tissue Antigens* (1988) 31(2): 90-97.

Kolstad, A, B Johansen and K Hannestad. Two HLA-DQ-specific human-human hybridoma antibodies (TrG6;TrC5) define epitopes also expressed by a transcomplementing hybrid DQ molecule (DQw7 alpha/DQw4 beta). *Hum Immunol* (1989b) 24(1): 15-29.

Koning, F, J Raghoebar, GM Schreuder, R Schuurman and H Bruning. A monoclonal antibody detecting an HLA-DQwl-related determinant. *Tissue Antigens* (1985) 26(2): 100-109.

Kosinski, S, U Hammerling and SY Yang. A human monoclonal antibody to an HLA-DRw53 (MT3)-like epitope on class II antigens. *Tissue Antigens* (1986) 28(3): 150-162.

Kramer, CSM, MEI Franke-van Dijk, KH Bakker, M Uyar-Mercankaya, GE Karahan, DL Roelen, et al. Generation and reactivity analysis of human recombinant monoclonal antibodies directed against epitopes on HLA-DR. *Am J Transplant* (2020)

Lardy, NM, AR van der Horst, EM van den Berg-Loonen, RE Bontrop and LP de Waal. Fine specificity of the alloantiserum MSD-51: epitope mapping of HLA-DRw53 determinants. *Hum Immunol* (1991) 32(1): 65-71.

Laundy, GJ, BA Bradley, BM Rees, M Younie and JM Hows. Incidence and specificity of HLA antibodies in multitransfused patients with acquired aplastic anemia. *Transfusion* (2004) 44(6): 814-825.

Layet, C, B Kahn-Perles, P Pontarotti, P Ferrier, J Sire and FA Lemonnier. Creation of an HLA-A2/HLA-Aw69 alloantigenic determinant on an HLA-A3 molecule by site-directed mutagenesis. *J Immunol* (1987) 138(7): 2197-2201.

Löffler, D, M Welschof, SF Goldmann and A Wölpl. Recognition of HLA-DR1/DRB1*0101 molecules presenting HLA-A2 derived peptides by a human recombinant antibody, Fab-5 A1. *Eur J Immunogenet* (1998) 25(5): 339-347.

Loh, MT, SH Chan and EC Ren. A monoclonal antibody with specificity to the HLA-DR1 and -DR51 antigens. *Tissue Antigens* (1993) 42(2): 100-104.

Lomago, J, L Jelenik, D Zern, J Howe, J Martell, A Zeevi, et al. How did a patient who types for HLA-B*4403 develop antibodies that react with HLA-B*4402? *Hum Immunol* (2010) 71(2): 176-178.

Lutz, CT, KD Smith, NS Greazel, BE Mace, DA Jensen, JA McCutcheon, et al. Bw4-reactive and Bw6-reactive antibodies recognize multiple distinct HLA structures that partially overlap in the alpha-1 helix. *J Immunol* (1994) 153(9): 4099-4110.

Madrigal, JA, H Ikeda, SG Marsh, JM Heyes, LJ Kennedy, J Trowsdale, et al. Epitope mapping of an HLA-DR-specific monoclonal antibody produced by using human-mouse transfectant cells. *J Immunol* (1989) 143(12): 4084-4089.

Maeda, H, R Hirata, H Kambayashi, F Koning and GM Schreuder. Multiple epitopes on a single DQ molecule from the DQw3-carrying haplotypes. *Tissue Antigens* (1986) 28(3): 136-145.

Marrari, M and RJ Duquesnoy. Why can sensitization by an HLA-DR2 mismatch lead to antibodies that react also with HLA-DR1? *Hum Immunol* (2009) 70(6): 403-409.

Marsh, SG and JG Bodmer. HLA-DR and -DQ epitopes and monoclonal antibody specificity. *Immunol Today* (1989) 10(9): 305-312.

Marsh, SGE, JH Moses and JG Bodmer. HLA class II sequence polymorphism detectable by serology. In: K Tsuji, M Aizawa and T Sasazuki, editors. *HLA 1991 - Proceedings of the Eleventh International Histocompatibility Workshop and Conference – Volume 1*. Oxford: Oxford University Press (1992). p. 610-619.

Marshall, WH, S Drover, D Codner, J Gamberg, MD Copp, HW Liu, et al. HLA-DP epitope typing using monoclonal antibodies. *Hum Immunol* (1998) 59(3): 189-197.

Marshall, WH, S Drover, H Mervart, HB Younghusband and H Liu. Production of a series of monoclonal antibodies to polymorphisms of HLA-DP. In: K Tsuji, M Aizawa and T Sasazuki, editors. *HLA 1991 - Proceedings of the Eleventh International Histocompatibility Workshop and Conference – Volume 2*. Oxford: Oxford University Press (1992). p. 415-417.

Matsuki, K, T Juji and M Satake. A novel HLA class II alloantigen detected by an alloantiserum TK2. *Hum Immunol* (1987) 18(3): 247-256.

Maurer, D and J Gorski. Transfer of polymorphic monoclonal antibody epitopes to the first and second domains of HLA-DR beta-chains by site-directed mutagenesis. *J Immunol* (1991) 146(2): 621-626.

Maurer, D, JA Madrigal and J Gorski. Thr77 controls a public antibody epitope on HLA-DRB1. In: K Tsuji, M Aizawa and T Sasazuki, editors. *HLA 1991 - Proceedings of the Eleventh International Histocompatibility Workshop and Conference – Volume 2*. Oxford: Oxford University Press (1992). p. 404-406.

Mazzoleni, O, A Longo, G Angelini, M Colonna, N Tanigaki, L Delfino, et al. Human monoclonal antibody MP8 detects a supertypic determinant encoded by DPB alleles DPB2.1, DPB3, DPB4.2, DPB8, DPB9, DPB10, and DPB14. *Immunogenetics* (1989) 30(6): 502-505.

Mulder, A, C Eijsink, MG Kester, ME Franke, MJ Kardol, MH Heemskerk, et al. Impact of peptides on the recognition of HLA class I molecules by human HLA antibodies. *J Immunol* (2005) 175(9): 5950-5957.

Mulder, A, M Kardol, J Blom, WB Jolley, CJ Melief and H Bruning. A human monoclonal antibody, produced following in vitro immunization, recognizing an epitope shared by HLA-A2 subtypes and HLA-A28. *Tissue Antigens* (1993) 42(1): 27-34.

Mulder, A, M Kardol, J Regan, R Buelow and F Claas. Reactivity of twenty-two cytotoxic human monoclonal HLA antibodies towards soluble HLA class I in an enzyme-linked immunosorbent assay (PRA-STAT). *Hum Immunol* (1997) 56(1-2): 106-113.

Mulder, A, MJ Kardol, CM Uit het Broek, J Tanke-Visser, NT Young and FH Claas. A human monoclonal antibody against HLA-Cw1 and a human monoclonal antibody against an HLA-A locus determinant derived from a single uniparous female. *Tissue Antigens* (1998) 52(4): 393-396.

Muller, C, S Liangru, M Schneider, A Ziegler and P Wernet. A cytotoxic monoclonal IgM antibody (Tu 101) directed against an antigenic determinant shared between the HLA-A allospecificities A2 and A28. *Hum Immunol* (1983) 6(4): 189-197.

Nakayama, T, K Ogasawara, H Ikeda, H Kunikane, M Kasahara, N Ishikawa, et al. A cytotoxic monoclonal antibody (HU-39) that detects DRw8 + DRw12. *Hum Immunol* (1987) 19(2): 117-126.

Navarette, C, C Brown, P de Lange and GMT Schreuder. 12th International Histocompatibility Workshop HLA class II monoclonal antibodies study. In: D Charron, editor. *HLA: Genetic diversity of HLA: Functional and Medical Implications Volume 1 - Proceedings of the Twelfth International Histocompatibility Workshop*. Paris, France: EDK (1997). p. 11-17.

Nelson, K, J Bodmer, A Martin, C Navarette and DM Strong. Micro EIA and Monoclonal Antbodies. In: B Dupont, editor. *Immunobiology of HLA Volume I: Histocompatibility Testing*. New York: Springer-Verlag (1989). p. 292-301.

Nordwig, H, WW Kwok and JP Johnson. Identification of the amino acid residues contributing to monoclonal antibody-defined DQw1 epitopes. *Hum Immunol* (1991) 31(2): 81-85.

Ogasawara, K, M Kasahara, T Nakayama, N Ishikawa, H Kunikane, S Hawkin, et al. A monoclonal antibody recognizing a polymorphic determinant on HLA-DR antigens except DR7 and DRw9. *Tissue Antigens* (1985) 26(5): 344-347.

Petersen, JS and T Dyrberg. Production of epitope specific monoclonal IgG antibodies to HLA class II molecules by combining in vivo and in vitro immunization. *J Immunol Methods* (1992) 151(1-2): 15-26.

Pistillo, MP, U Hämmerling, B Dupont and GB Ferrara. In vitro production of a human HLA alloantibody of restricted specificity (DQw2) via Epstein-Barr virus transformation. *Hum Immunol* (1986) 15(1): 109-117.

Pistillo, MP, RW Karr, E Klohe, O Mazzoleni and GB Ferrara. Use of transfectants to identify polymorphic epitopes recognized by human monoclonal antibodies. In: K Tsuji, M Aizawa and T Sasazuki, editors. *HLA 1991 - Proceedings of the Eleventh International Histocompatibility Workshop and Conference – Volume 2*. Oxford: Oxford University Press (1992). p. 413-415.

Pistillo, MP, O Mazzoleni, L Kun, M Falco, PL Tazzari and GB Ferrara. Production of two human hybridomas secreting antibodies to HLA-DRw11 and--DRw8+w12 specificities. *Hum Immunol* (1991) 31(2): 86-93.

Pistillo, MP, V Sguerso and GB Ferrara. High yields of anti-HLA human monoclonal antibodies can be provided by SCID mice. *Hum Immunol* (1992) 35(4): 256-259.

Radka, SF, KA Nelson and JV Johnston. HLA-DQw3-related determinants: analysis of subunit and spatial relationships. *Hum Immunol* (1989) 25(4): 225-236.

Radka, SF, SJ Stewart and SA Smith. Analysis of HLA-DQ molecules with a monoclonal antibody detecting a DQ polymorphism absent from DQW1 homozygous cells. *Hum Immunol* (1985) 14(3): 206-219.

Sadler, AM, JM Heyes, SG Marsh, P Krausa, GE Reynolds and JG Bodmer. The monoclonal antibody TAL16.1 recognizes the aspartic acid residue at position 70 in DRB gene products. *Tissue Antigens* (1993) 41(1): 42-46.

Sánchez, B, O de la Calle, J Yélamos, I Aguilera, F Sánchez, V Dessi, et al. A human monoclonal antibody reacting against HLA-DQ1-, DQ4-, and a subset of DQ7-bearing cells. *Hum Immunol* (1993) 36(2): 81-90.

Schreuder, GM, H Maeda, F Koning and J D'Amaro. TA10 and 2B3, two new alleles in the HLA-DQ region recognized by monoclonal antibodies. *Hum Immunol* (1986) 16(2): 127-136.

Tambur, AR, JR Leventhal, JJ Friedewald and DS Ramon. The complexity of human leukocyte antigen (HLA)-DQ antibodies and its effect on virtual crossmatching. *Transplantation* (2010) 90(10): 1117-1124.

Taylor, CJ, L Ugozzoli, N Tanigaki, R Tosi, M Bunce, A Ting, et al. Antigen Sociey #29 Report (DRw52). In: B Dupont, editor. *Immunobiology of HLA Volume I: Histocompatibility Testing* New York: Springer-Verlag (1989). p. 273-275.

Thammanichanond, D, W Parapiboon, T Mongkolsuk, S Worawichawong, C Tammakorn and P Kitpoka. Acute Antibody-Mediated Rejection by De Novo Anti-HLA-DPβ and -DPα Antibodies After Kidney Transplantation: A Case Report. *Transplant Proc* (2018) 50(8): 2548-2552.

Tongio, MM, I Doxiadis, M Laforet, GMT Schreuder, N Froelich, G Semana, et al. 12th International Histocompatibility Workshop HLA class I monoclonal antibodies study. In: D Charron, editor. *HLA: Genetic diversity of HLA: Functional and Medical Implications Volume 1 - Proceedings of the Twelfth International Histocompatibility Workshop*. Paris, France: EDK (1997). p. 7-11.

Tongio, MM, E Van den Berg-Loonen, JD Bignon, D Chandanayingyong, A Dormoy, T Eiermann, et al. HLA-DP detected by serology. In: D Charron, editor. *HLA: Genetic diversity of HLA: Functional and Medical Implications Volume 2 - Proceedings of the Twelfth International Histocompatibility Conference*. Paris, France: EDK (1997). p. 135-138.

Van den Berg-Loonen, EM, JD Bignon, E Du Toit, EM Pitchappan, J Loon, DP Singal, et al. AHS#11: HLA-DR2 (DR15, DR16), DR51. In: D Charron, editor. *HLA: Genetic diversity of HLA: Functional and Medical Implications Volume 1 - Proceedings of the Twelfth International Histocompatibility Workshop*. Paris, France: EDK (1997). p. 91-95.

Viken, HD, G Gaudernack and E Thorsby. Characterization of a monoclonal antibody recognizing a polymorphic epitope mainly on HLA-DPw2 and DPw4 molecules. *Tissue Antigens* (1989) 34(4): 250-259.

Viken, HD, G Paulsen, S Drover, WH Marshall, LM Sollid, G Gaudernack, et al. Influence on antibody recognition of amino acid substitutions in the cleft of HLA-DQ2 molecules. Suggestive evidence of peptide-dependent epitopes. *Hum Immunol* (1995a) 44(2): 63-69.

Viken, HD, G Paulsen, LM Sollid, KE Lundin, GE Tjønnfjord, E Thorsby, et al. Characterization of an HLA-DQ2-specific monoclonal antibody. Influence of amino acid substitutions in DQ beta 1*0202. *Hum Immunol* (1995b) 42(4): 319-327.

Viken, HD, AB Thoresen, E Thorsby and T Hansen. The cytotoxic HLA-DQ3 reactive human hybridoma antibody 4166 that may distinguish DQ7 + 8 from DQ9. *Hum Immunol* (1995c) 42(4): 281-288.

Viken, HD, E Thorsby and G Gaudernack. Characterization and epitope mapping of four HLA class II reactive mouse monoclonal antibodies using transfected L cells and human cells transfected with mutants of DQB1*0302. *Tissue Antigens* (1995) 45(4): 250-257.

Vilella, R, J Yague and J Vives. Monoclonal antibody against HLA-Aw32 + A25. Is HLA-Aw32 an allele with no unique antigenic determinant? *Hum Immunol* (1983) 6(1): 53-62.

Vitale, M, MP Pistillo, PL Tazzari, M Falco, PF Sun, S Mantero, et al. Production and characterization of murine monoclonal antibodies recognizing HLA-DQ polymorphisms obtained by immunizing mice with transfected L cells. *Hum Immunol* (1992) 34(2): 126-134.

Wölpl, A, T Halder, H Kalbacher, H Neumeyer, K Siemoneit, SF Goldmann, et al. Human monoclonal antibody with T-cell-like specificity recognizes MHC class I self-peptide presented by HLA-DR1 on activated cells. *Tissue Antigens* (1998) 51(3): 258-269.

Young, JA, J Lindsay, JG Bodmer and J Trowsdale. Epitope recognition by a DP alpha chain-specific monoclonal antibody (DP11.1) is influenced by the interaction between the DP alpha chain and its polymorphic DP beta chain partner. *Hum Immunol* (1988) 23(1): 37-44.

Youngs, D. DP alloantibodies. *ASHI Quarterly* (2004):60-62.

Youngs, D, P Warner, M Gallagher and I Gimferrer. New DQA1 allele specific antibody against epitope 2D (an exon 1 encoded amino acid). Considerations for alleles under the same P-group designation. *Transpl Immunol* (2018) 51: 32-39.
